# A State-of-the-Art Review on Innovative Geopolymer Composites Designed for Water and Wastewater Treatment

**DOI:** 10.3390/ma14237456

**Published:** 2021-12-04

**Authors:** Ismail Luhar, Salmabanu Luhar, Mohd Mustafa Al Bakri Abdullah, Rafiza Abdul Razak, Petrica Vizureanu, Andrei Victor Sandu, Petre-Daniel Matasaru

**Affiliations:** 1 Department of Civil Engineering, Shri Jagdishprasad Jhabarmal Tibrewala University, Rajasthan 333001, India; jprraj2017@gmail.com; 2 Center of Excellence Geopolymer and Green Technology (CEGeoGTech), Universiti Malaysia Perlis (UniMAP), Perlis 01000, Malaysia; rafizarazak@Unimap.edu.my; 3 Frederick Research Center, P.O. Box 24729, Nicosia 1303, Cyprus; 4 Department of Civil Engineering, Frederick University, Nicosia 1036, Cyprus; 5 Faculty of Materials Science and Engineering, Gheorghe Asachi Technical University of Iasi, D. Mangeron 41, 700050 Iasi, Romania; 6 Romanian Inventors Forum, St. P. Movila 3, 700089 Iasi, Romania; 7 National Institute for Research and Development in Environmental Protection INCDPM, Splaiul Independentei 294, 060031 Bucuresti, Romania; 8 Faculty of Electronics, Telecommunications and Information Technology, Technical University “Gheorghe Asachi”, Carol I Bvd, nr. 11 A, 700506 Iasi, Romania; dmatasaru@etti.tuiasi.ro

**Keywords:** wastewater, geopolymers, geopolymer composite, water treatment, geopolymer adsorbent, heavy metal removal, nutrient recovery

## Abstract

There is nothing more fundamental than clean potable water for living beings next to air. On the other hand, wastewater management is cropping up as a challenging task day-by-day due to lots of new additions of novel pollutants as well as the development of infrastructures and regulations that could not maintain its pace with the burgeoning escalation of populace and urbanizations. Therefore, momentous approaches must be sought-after to reclaim fresh water from wastewaters in order to address this great societal challenge. One of the routes is to clean wastewater through treatment processes using diverse adsorbents. However, most of them are unsustainable and quite costly e.g. activated carbon adsorbents, etc. Quite recently, innovative, sustainable, durable, affordable, user and eco-benevolent Geopolymer composites have been brought into play to serve the purpose as a pretty novel subject matter since they can be manufactured by a simple process of Geopolymerization at low temperature, lower energy with mitigated carbon footprints and marvellously, exhibit outstanding properties of physical and chemical stability, ion-exchange, dielectric characteristics, etc., with a porous structure and of course lucrative too because of the incorporation of wastes with them, which is in harmony with the goal to transit from linear to circular economy, i.e., “one’s waste is the treasure for another”. For these reasons, nowadays, this ground-breaking inorganic class of amorphous alumina-silicate materials are drawing the attention of the world researchers for designing them as adsorbents for water and wastewater treatment where the chemical nature and structure of the materials have a great impact on their adsorption competence. The aim of the current most recent state-of-the-art and scientometric review is to comprehend and assess thoroughly the advancements in geo-synthesis, properties and applications of geopolymer composites designed for the elimination of hazardous contaminants viz., heavy metal ions, dyes, etc. The adsorption mechanisms and effects of various environmental conditions on adsorption efficiency are also taken into account for review of the importance of Geopolymers as most recent adsorbents to get rid of the death-defying and toxic pollutants from wastewater with a view to obtaining reclaimed potable and sparkling water for reuse offering to trim down the massive crisis of scarcity of water promoting sustainable water and wastewater treatment for greener environments. The appraisal is made on the performance estimation of Geopolymers for water and wastewater treatment along with the three-dimensional printed components are characterized for mechanical, physical and chemical attributes, permeability and Ammonium (NH_4_^+^) ion removal competence of Geopolymer composites as alternative adsorbents for sequestration of an assortment of contaminants during wastewater treatment.

## 1. Introduction

“Water is life” for all breathing organisms. Incredibly, our mother planet earth is 70% covered by water; however, we have merely 3% freshwater useable by lives breathing on it! Apprehensively, over recent decades, a signaling boost in water demand has cropped up as a major predicament of “water scarcity”, which is directly linked to “water stress” or “water crisis”, i.e., the lack of fresh, clean and potable water resources in order to meet the standard and rising exigency. Staggeringly, the emerging clean water crisis is turning out to be an international concern since it is impacting 785 million persons and 1.1 billion are those who lack access to water, while 2.7 billion are experiencing water shortages. In accordance with the estimation, by 2025, almost 1800 million people will be living in the areas having absolute scarcity of water, while about 66% of the world population will pass through the stressful conditions [1]. Shockingly, the other estimation assesses that more than 40% of the global population will have to live in severe water scarcity areas by 2050 [2]. This happening is associated with the mushrooming international population and the eco-unfriendly industrial growth that has accelerated the incredible demand for water and also the pollution of water to unprecedented echelons. Of which, the latter mounds the pressure on the accessible sources to deal with the demand. For this reason, water and wastewater treatment are of prime significance as they can enhance the supply of water. Recently, the World Economic Forum report has also regarded water scarcity as the gravest concern for our society. Even in nations with enough water resources, water dearth is not uncommon. Consequently, the EU has publicized the Water Framework Directives for the adoption of specific legislation to handle the situation [1,2].

On the other hand, the pollution of water is one of the most severe environmental concerns as it carries heavy metals and lethal pollutants with it, which are difficult to biodegrade. The terrifying organic pollutants encompass plasticizers, phenols, poly-nuclear aromatic hydrocarbons (PAHs), polychlorinated biphenyls (PCBs), poly-brominated diphenyl ethers (PBDEs), pesticides and drug residues, while the chief inorganic contaminants are toxic metals together with nutrients, such as nitrate and phosphate [3]. Heavy metal cations are regarded as one of the most perilous contaminants in water and wastewater because of their very high toxicity and ability to accumulate in living tissues [4,5]. These contaminants, when entering the human body via the food chain, accumulate and can cause chronic poisoning [6]. 

Cadmium (Cd) and Lead (Pb) are the most noxious heavy metals responsible for numerous diseases [7]. Under these terrible situations, an approach to get clean water from wastewater by removing its enclosed polluting agents seems to be a momentous viable solution, helpful for both predicaments of a scarcity of water, as well as wastewater management. Simply speaking, “wastewater” is the water that has been used at least once or more and gets polluted. It is generated from the release of an assortment of pollutants from domestic, municipal, industrial, businesses, etc., sectors into freshwater bodies directly or indirectly. It encloses human wastes, oils, grease, soaps, food scraps, diverse chemicals, toxic materials, storm runoff (such as debris, woods, rocks, plastics, at times hazardous heavy metals), dissolved gases (such as hydrogen sulfide (H_2_S)) bacteria, viruses, disease-causing pathogens, a few discarded pharmaceuticals, dyes, surfactants, pesticides, personal hygiene care items and even dead animals, etc. However, nature owns an astonishing aptitude to deal with smaller quantities of contaminants in wastewater, but it would be overwhelmed if the daily generation of billions of gallons of wastewater and sewage are not subjected to systematic treatment prior to release back to the environment. It is a universal fact that water is a widespread solvent; hence, it is considered a noteworthy source of diverse pollutions and infections. The world health organization (WHO) in its alarming account warned that around 80 % of diseases are assigned to pollution of water since the stated WHO standards are not being followed appropriately and stringently [8].

Clean water is highly needed for life and is also a great playground for us all and, therefore, wastewater should be treated methodically in order to have potable water and advantages for the environment, the health of living beings, ecology, water bodies, etc. If wastewater management is not made systematic, it will negatively impact land populations, aquatic lives, fauna and flora, wildlife; it will contaminate drinking and surface water; lead to oxygen (O_2_) depletion, beach closures, limitations on recreational water use, restrictions on fish and shellfish harvesting, etc. The decaying of organic matter and debris consume the dissolved O_2_ in water bodies, so aquatic biota cannot survive. Analogously, the chlorine compounds and inorganic chloramines are toxic to algae, fish and invertebrates living in water bodies. An excessive presence of nutrients of phosphorus (P) and nitrogen (N), including ammonia (NH_3_), carried along by wastewater can cause “excessive fertilization or eutrophication” of receiving waters, which may be noxious to organisms existing in the water. Not only that, they encourage unwarranted extreme plant growth, trim down O_2_, damage the spawning grounds, modify the habitat and lead to a turndown of specific species. With a view to deal with water scarcity, wastewater can be converted to potable through different treatments with the use of absorbents to remove pollutants, such as heavy metals in the form of lead (Pb), cadmium (Cd), chromium (Cr), cobalt (Co), arsenic (As), mercury (Hg), radionuclide [9], etc., which can have chronic and acute toxic influences, and can also bio-accumulate [10]. Thus, the subsequent reuse of wastewater can offer a significant rise in clean water supply, addressing the setback of water paucity. Wastewater treatment includes a number of methods, e.g., heavy metal removal can be made possible through chemical precipitation, coagulation-flocculation, floatation and reverse osmosis [11]. Ion-exchange, adsorption, supercritical fluid extraction, advanced oxidation course and membrane bio-reactors, filtration, electro-dialysis, microbial system, electro-chemical procedures, Fenton oxidation, photo-degradation, etc. [12,13,14], are utilized for the subtraction of heavy metals, dyes and other contaminants from wastewater, each has their own pros and cons. The process’s efficiency, eco-friendliness and financial viability restrict the use of the conventional techniques mentioned. However, adsorption has proved to be most useful and most expansively employed on account of not only its simplicity and efficiency but also for its low cost amongst all the above-listed routes for the exclusion of heavy metals, dyes and other contaminants (viz., zeolites, activated carbon, resins, fly ash, chitosan, alumina and silica, as well as diverse materials, such as ZnO nanoparticles, CuO nanoparticles, active graphene oxide, boron nitride, etc. [15,16,17,18]) represent higher competence levels, but their higher production cost is a hindrance to their across-the-board application. 

Therefore, in order to address all of the aforementioned predicaments, an orderly treatment of wastewater is highly essential to follow for sustainable eco-system, human health, the breathability of living beings, financial productivity in the course of recovery of valuable materials, etc. The key objective of the wastewater treatment process is to get rid of as much of the suspended solid contaminants as possible from wastewater and convert it into the remaining water, known as “effluent”, which is returned to the water cycle in the environment. Once back in the water cycle, the effluent creates the standard impact on the eco-system or is reused for an assortment of objectives, known as “water reclamation”. The treatment process performed in a wastewater treatment plant depends upon the types of wastewater, such as domestic or municipal wastewater or sewage treatment plant, or industrial wastewater treatment plant by and large subsequent to a few forms of prior treatments; along with the agricultural wastewater treatment plants and leachate treatment plants. On the whole, the procedures in practice include phase separation, such as sedimentation, the biological and chemical course of actions, e.g., oxidation or polishing. The primary by-product generated from wastewater treatment plants is a kind of sludge that is normally treated in the same or a different wastewater treatment plant. If an anaerobic treatment progression is utilized, then biogas (Methane—CH_4_) may be an additional by-product. The primary processes of wastewater treatment include screening, pumping, aerating, and elimination of organic matters, i.e., sludge, removal of scum consisting of grease, oils, plastics and soap; filtration, usually through sand by the action of gravity to remove all the bacteria, which decreases the turbidity and color, takes away odors, mitigates iron quantity and removes most other solid particles that remained. Sometimes, in order to remove organic particles, filtering through carbon particles is followed. Ultimately, with a view to kill the bacteria, the wastewater flows into a “chlorine contact” tank, where the chemical chlorine is added. Thus, the effluent, i.e., the treated water, is then discharged into a local river or the ocean. “Wastewater Residuals” is one more portion of wastewater treatment that deals with the solid waste materials, which are kept for 20 to 30 days in larger enclosed and heated tanks known as “digesters”, whereby the bacteria digest the material, resulting in a reduction in its volume, odors and removal of disease-causing organisms. The end-product is, for the most part, dumped into landfills; however, at times, it is found useful as fertilizer.

Accordingly, wastewater can be regarded as a resource that is so valuable that no throwing away is acceptable, particularly on our globe where there exists an increasing water scarcity. For this reason, nowadays, the reuse of treated wastewater and water conservation are increasingly becoming more significant. Appreciably, the reclamation of wastewater frees up freshwater, which can be utilized at another place where it is needed, for example, for drinking purposes, thus conserving drinking water and offers water for irrigational and industrial applications. Incredibly, even astronauts of the International Space Station drink urine following reclamation because, in outer space, water is at a premium, and therefore, not even a single drop is to be squandered.

On the other hand, quite recently, an innovative geopolymer technology has attracted world concrete researchers mostly due to its sustainable, durable and affordable approaches. The hitch of lack of sustainability in several other common practices of treatment technologies, viz., adsorption, ion exchange, chemical treatment, coagulation, etc. [11,19]. Among the list, adsorption gives the impression as the most promising, efficient and within one’s means for wastewater treatment, particularly using geopolymer adsorbents [20]. The “geo-synthesis” of geopolymers involves the activation of rich alumina-silicates with strong alkali activators at low temperatures and atmospheric pressure only, popularly known as “geopolymerization” in an alkaline medium. Geopolymers are amorphous to semi-crystalline in structures, which are produced by an exothermal chemical reaction between silica and alumina-rich precursors with alkaline activators in an alkaline medium, keeping the temperature low at atmospheric pressure with low energy consumption, representing a lower carbon footprint. Since the chemical structure of geopolymers is comprised of a negatively-charged alumina-silicate framework, the charge-balancing can be exchanged with the cations present in the activator solution, making them suitable for employing as valuable adsorbents for wastewater treatment. 

Geopolymers are versatile materials that have created a center of attention in scores of environmental applications, for instance, they have been investigated as “Sorbents” for wastewater treatment which is assigned to the key drivers in form of excellent mechanical and chemical stability together with a relatively simple, low operational energy, and lower carbon footprint bearing production process for this emerging interest [20]. Practically, Geopolymer sorbents can conveniently be installed in pipes or columns, whereby the water is pumped by means of the lattice structure to interact with the active surface sites enclosing cations like Na^+^, etc. which are exchangeable [20]. Accordingly, the geopolymers are competent enough to offer viable alternatives for competing materials for application for wastewater treatment in form of conventional ceramics, synthetic zeolites, or polymeric components in virtue of not only cost but also in performance and significantly in eco-system impacts. In the interest to prevent eutrophication, the legislative need for the elimination of nitrogen present in the wastewaters generated from municipal and industrial operations is turning out to be increasingly widespread [19,20]. 

The objective of the present review manuscript is not only to lend a hand to comprehend the significance of geopolymers as most modern adsorbents to eliminate perilous and noxious pollutants from wastewater in order to get potable and clean reclaimed water for reuse, extending relief to the gigantic impasse of a scarcity of water but also encourage sustainable water and wastewater treatment for green eco-systems. The assessment is to be made for the performance evaluation of geopolymers for water and wastewater treatment.

Web of Science, ScienceDirect, ResearchGate, SpringerLink, and other databases were used to find out more about novel geopolymer composites for water and waste water treatment. The keywords “geopolymer, waste water treatment, water treatment, and geopolymerization” were identified in the Scopus database. The goal of this study is to highlight current research and application status in the domains of geopolymer -water and wastewater treatment technology. For reference, the related references provided in the literature were also used.

## 2. Introduction of Geopolymers

Geopolymers are a class of ceramic-like inorganic polymers analogous to the Felspathoid family of minerals in their mineralogical attributes, which can be developed at a considerably low temperature of below 100 °C and merely at atmospheric pressure by means of an exothermal process of geopolymerization, i.e., through a polycondensation reaction obtained by alkali activation of an alumina-silicate source as a precursor, such as alumina-silicate minerals [21] (metakaolin) or industrial wastes, e.g., fly ash, ground granulated blast furnace slag (GGBFS), coal gangue, red mud, etc., and alkali-activating solutions as activators, viz., sodium hydroxide (NaOH), sodium water glass (Na_2_SiO_3_), etc., in this process, which is also known as “geo-synthesis”, formulating amorphous polymers possessing the chains or networks of mineral molecules linked to covalent bonds [22]. Besides, acid-based activators, such as phosphoric acid, and aluminum phosphate-based activators have been discovered to be a viable alternative to alkali-based activators. According to research, geopolymer produced by phosphate-based activators has better mechanical and microstructural properties than geopolymer produced by alkali-based activators, and the absence of alkali ions and an increase in bridging oxygen in the former geopolymer than the latter are the reasons for superior performance [23].

Geopolymerization occurs in three separate but interrelated stages—firstly, the dissolution, i.e., alumina-silicate precursors, get dissolved in an alkali activator solution in order to form free AlO_4_ and SiO_4_ tetrahedral units; secondly, the condensation, whereby two tetrahedral units form a long chain because of the condensation reaction kinetics; and thirdly, the poly-condensation reactions stage wherein the reactions among the long chains to produce gel-like materials, which are mainly amorphous N–A–S–H (sodium–alumino–silicate–hydrate) and C–S–H (calcium-silicate-hydrate) or the Al-substituted C–A–S–H (Calcium–Aluminium–Silicate–Hydrate) gel in their form [24]. Following the reaction kinetics, the rock-hardened rigid material developed which contains an amorphous three dimensional (3D) structure comprising of AlO_4_— and SiO_4_-tetrahedra linked alternatively by sharing oxygen (O) atoms, coined as poly-sialates by French scientist Joseph Davidovits [24]. The motive behind the use of alkali silicate is three-fold, i.e., the silicate molecules are taking part in the formulation of the poly-sialate, the alkali portion existing in the solution causes the cleavage of the alumina-silicate precursor; and this activator solution is also the source of the metal cations for charge balancing which is an indispensable precondition. Thus, the negative charge of tetrahedral-coordinated Al-atoms inside the network is balanced by cations sourced from the activating solution. The vital phase is of hydrated sodium alumina-silicates (N–A–S–H) gel, which is a flowable paste, and it is a network built of silicon (Si) and aluminum (Al) tetrahedra, linked together through oxygen bridges. The oxygen tetrahedra holding Al^3+^ ions hold a negative charge that is neutralized by an alkali metal cation. Consequently, geopolymers are the result of a mineral polycondensation reaction, which involves dissolution and condensation. Cations of Na^+,^ K^+^, Li^+^, Ca^2+^, etc., must be present in the voids of the poly-sialate with a view to balancing the negative charge of the tetravalent aluminum (Al).

The empirical formula as per Davidovits for a poly-sialate can be portrayed as follows [24]:

Mn [(-SiO_2_)z-AlO_2_]n.wH_2_O, (1)

whereby M—an alkali cation (Na^+^, K^+^, Li^+^, etc.); n—degree of polycondensation; and z—an atomic ratio of Si:Al, which may be equivalent to 1 to 32.

Poly-sialates will form an amorphous or semi-crystalline matrix at ambient temperatures and low water contents relying upon the reaction conditions. The atomic ratios of Si:Al in the poly-sialates determine the physical characteristics and uses of the end-product. The atomic ratio of Si:Al plays an important role in the diverse final products. The condensation can take place, even at room temperature, and for this reason, geopolymers are frequently considered cementitious materials. Even so, at elevated temperatures, crystalline phases develop and sintering reactions result in the development of ceramic products. Evidentially, the geopolymer system exhibited enhanced strength, durability, resistance against acid attack, thermal and fire in comparison to most cementitious matrices and Portland cement/concrete. On top of that, they are user and eco-friendly with low operational energy. That is why their numerous potential applications are proposed. Fly ash-based geopolymers are identified as an eco-friendly, purer and chemically homogeneous replacement of ordinary Portland cement (OPC) while those metakaolin based are being further employed in more significant technical applications viz., encapsulation or immobilization of radioactive nuclear wastes. Recently, a novel potential application of geopolymers for wastewater treatment was found to be eye-catching. Admirably, this approach is advantageous because of its higher strength, low permeability, user and eco-benevolent nature, together with fire-resistance and improved dimensional stability [22,25]. Several studies are directed in searching for low-cost raw materials appropriate for zeolite synthesis [25].

The chemistry of geopolymers and the reaction kinetics of geopolymerization have ended the days of high temperatures and intense energy processes to obtain the materials of ceramic-like structure, demonstrating equivalent properties as a conventional system. Consequently, no more energy-intensive and elevated temperature reactions are necessary anymore, as found in that of the cement system. In fact, the investigations have proven that the geopolymers are zeolite-like structures comprising [SiO_4_]^−4^ and [AlO_4_]^−5^ tetrahedra linked by oxygen atoms, wherein the negative charges of the [AlO_4_]^−5^ tetrahedra are balanced by alkali cations accessible from either the activator or precursors. Nevertheless, the geopolymers are amorphous or semi-crystalline, with heterogeneous inner micro-pores, such as zeolites [22,23,24,25].

Even so, the application of geopolymers as adsorbents is quite recent, and the breathing literature is, therefore, also limited [26,27]. The study reveals that both Metakaolin-based and coal fly ash-based geopolymers [27] have displayed an uptake aptitude for lead (Pb) more or less 100 and 80 mg/g, in that order. That simply means that the latter exhibits the luminous practicability of utilizing geopolymers as heavy metals adsorbents. The middle-of-the-road investigations have thrown lights upon the incorporation of diverse wastes used as precursors in the geopolymer’s production, such as fly ash, metakaolin, blast furnace slag, etc. Extensively, the industrial by-product of Coal fly ash has been regarded as an alumina-silicate source. Geopolymer composites were produced with a view to enhance the strength of the original material by supplementing chamotte by Musil et al. [28], whereas Mohseni [29] and Noushini et al. [30] used a mix of polypropylene along with other synthetic fibers.

Additionally, Saafi et al. [31] and Yan et al. [32] have employed a mixture of carbon nanotubes and graphene. As the promising adsorbents for wastewater treatment, porous geopolymers, were formulated, their performance is confined to their inherent definite surface area and pore structure. It might be enhanced by using pore-forming additions that can optimize the porous structure of geopolymers from the view of a composite. The coal gangue—a by-product generated during the coal mining process, which is not only a significant resource for the manufacturing of ceramic, cement, and construction materials [33,34] but also portrayed by a layered structure made up of the silicate, promising the admirable adsorption attributes subsequently to correct modification. Yan et al. [35,36,37] made obvious the good-quality adsorption characteristics of microspheres of coal gangue that makes them competent environmental adsorbents: the adsorption potential of methylene blue on microspheres of coal gangue can reach ~30 mg/g. The microspheres of coal gangue might be employed to manufacture porous geopolymer composites, which can be employed for wastewater treatment. The viability of the incorporation of wastes not only extends to reduce the environmental impact but also lowers the cost of production of geopolymers. A modest work is performed on the adsorption attributes of heavy metal ions. The structure of geopolymers makes their applications valuable for water remediation, i.e., photo-degradation of risky organic compounds, heavy metals adsorption, energy evolution/storage, antibacterial uses, etc. Their good adsorption performance, simple, reduced carbon footprint, low-temperature and lower operational energy process of geopolymerization and the cost-effectiveness and accessibility of wastes as raw materials have altogether made them popular and received momentous attention, from both researchers and industries, for water and wastewaters treatments [38,39,40,41].

## 3. Hazardous and Toxic Ions of Heavy Metals as Pollutants in Industrial Wastewater

The huge quantity of industrial wastewater is a solemn and enduring environmental setback on account of it carrying heavy metals with it [40]. Mostly the industrial products, such as heavy metals, pesticides, colorants, etc., can cause water and wastewater pollution resulting in eco-degradation. The non-biodegradable and extremely venomous heavy metals, such as cobalt (Co), lead (Pb), manganese (Mn), copper (Cu), cadmium (Cd), etc., are frequently found present as contaminants in a range of industrial wastewater, surface and subsurface waters. That is why it is highly essential to remove these metal ions from the industrial wastewater and water utilized for human use [41]. They are found to occur in wastewater generated from industries of the major source of heavy metal cations release, namely, electroplating and chemical-processing plants, as well as the coal washing, end-products of battery production, etc., posing progressively more grave pollution of ecology. The water pollution due to the inclusion of heavy metal ions is regarded as very perilous since these ions have a tendency to bio-accumulate in living organisms over time. The heavy metal ions cross the threshold of freshwater supplies through mining and industrial activities or from acid rain, which breaks down soils and liberates heavy metal ions into surface water bodies, viz., streams, lakes, rivers, and of course, sometimes sub-surface water too. For these reasons, it is extremely urgent and highly essential to remove them with a view of making the water clean through the treatment of enclosing wastewater [42,43], especially by means of the alternative affordable user and eco-friendly geopolymer adsorbents. By and large, the following heavy metals are found associated with wastewater, making the job of its management very challenging.

### 3.1. Lead (Pb)

Lead is one of the heavy metals, and it causes industrial pollution. Having a chemical symbol (Pb) and valences of ions as Pb^+2^ and Pb^+4^, it is one of the most widespread heavy metals found enclosed in industrial wastewaters, which is identified to be lethal for living beings [44]; hence, it is essential that its elimination from wastewaters is of prime significance since it is the most toxic of heavy metals answerable for so many diseases. The heavy metal contamination occurring from the industries has piloted to plentiful ecological and security crises. For instance, the accumulation of Pb^+2^ ions can harm different organs of human beings, viz., the kidneys, central nervous system, heart and the immune system; it can cause cancer and even death, besides causing physical developmental disorders in innocent children [45,46,47,48,49,50]. Hence, stringent standards are made for allowed Pb^+2^ concentrations in drinking and surface waters. For illustration, the World Health Organization (WHO), the European Union (EU) and India have narrowed the highest level of Pb^+2^ in drinking water down to 0.01 mg/L [51]. Consequently, significant notice is paid to the taking away of Pb^+2^ from the wastewater urgently through methods, namely, adsorption, ion exchange, membrane separation and precipitation [52,53,54]. However, adsorption is found with plenty of competitive advantages amongst all listed techniques, such as its simplicity, low price, and effectiveness, along with ease to design, regenerate and operate [55]. A variety of adsorbents, such as geopolymers, were investigated for the exclusion of Pb^+2^ [56]. For the most part, the studies on removing Pb^+2^ ions [57,58,59] are concentrated on the static removal mechanism. Nevertheless, the dynamic removal is found more in agreement with the practical usefulness [60]. Praiseworthily, geopolymers extend affordable, renewable and eco-benevolent widely-accepted adsorbents.

### 3.2. Cobalt (Co)

Cobalt (Co) is a chemical element having its ions as Co^+2^ and Co^+3^. It is an imperative trace element, and it is being extensively employed in plenty of industries as a part of diverse anthropogenic actions, such as mining, colorants in glass, paints, ceramics, discarded batteries, alloys manufacturing, petro-chemical, dye, metal industries, etc. [61]. By and large, it enters the environment from the eruptions of volcanoes, wildfires in forests, and overflow or leaching of the rainwater through natural rocks enclosing cobalt. It is a key metal ingredient of vitamin B12, which is essential for maintaining human health and in the treatment of anemia by helping to produce red blood cells, but it can be hazardous if consumed in extreme amounts. The permissible limits for cobalt to be present in drinking water, internal surface water, public sewers, irrigation water and industry are recommended as 0.01, 0.05, 0.5, 1.0 and 0.2 mg/L, respectively [62,63]. Beyond these limits, cobalt can cause a few discomforts, such as diarrhea, skin degeneration, vomiting, pneumonia, skin rashes, allergies, bone defects and impacts on organs, such as lungs resulting in asthma, etc. [64], and weight loss, as well as passing it on from mother to fetus during pregnancy and through milk during the period of breastfeeding. Therefore, the recommended standard level for cobalt proportion in drinking water, which is 2 μg/L, must be maintained [65]. In recent years, owing to these negative impacts of cobalt, a few endeavors to get rid of cobalt contamination from water and industrial wastewater have amplified with the help of geopolymer adsorbents in order to make them free from this hazardous pollutant. Therefore, it is necessary to remove Co^+2^ ions from the water and wastewater using geopolymers, kaolinite, activated carbon, palygorskite and sepiolite [63,66,67].

### 3.3. Chromium (Cr)

Chromium (Cr^+2^, Cr^+3^, Cr^+6^) ions are considered as heavy metal ions discharged from natural or industrial sources, such as tanneries, electroplating and other related chromium-generating industries [68]. In the environment, the chromium occurs primarily in two valence states, i.e., trivalent chromium (Cr^+3^) and hexavalent chromium (Cr^+6^), which have been spotted in wastewaters. However, Cr^+6^ is considered to be as much more risky and noxious than Cr^+3^ since it can damage human skin and internal organs directly. For that reason, the research studies have been concentrated on the mitigation of Cr^+6^ levels through cutback to the lesser venomous Cr^+3^. Nevertheless, Cr^+3^ is effortlessly re-oxidized to the perilous Cr^+6^ on its exposure to the oxidative environment [69], and so, the Cr^+3^ should also be eliminated.

### 3.4. Copper (Cu)

Copper is a chemical element with a symbol (Cu). It is an extensively utilized metal in copious industries. To name a few, leather tanning, mining, electroplating, brass manufacturing, petroleum refining (i.e., copper sweetening), coating, smelting [70]. Copper (Cu^+2^) ion is the chief heavy metal cation found in waste effluents generated from the electroplating industry [71]. Among the ions of Cu, the cupric (Cu^+2^) ion is commonly found in water as a more widespread oxidation state, forming complexes with carbonate (CO_3_^−2^) and hydroxide (OH^−1^) ions. The development of insoluble mineral Malachite with the chemical composition [Cu_2_(OH)_2_ CO_3_] in water is a key domineering feature of the level of free cupric (Cu^+2^) ions in an aqueous solution. Accordingly, drinking water gets contaminated with salts of Cu^+2^, negatively influencing living organisms with short and long-term impacts [72,73,74]. An excessive intake of Cu^+2^ ions pilots to accumulation in the human liver and can cause other solemn human health crises, such as mucosal irritation, damage to the renal or central nervous system [75], damage to the liver [76], since it is highly toxic to living beings and organisms even at a very low concentration. Therefore, taking into account the incessant eco-damage and the necessary need to remove these hazardous heavy metal ions from the water, as well as wastewater, the water and wastewater must be made clear by freeing them from these pollutants before their release into the environment and/or water bodies. It is recommended that the upper limit for the concentration of Cu^+2^ must not go beyond 2.0 mg/L in drinking water [77]. For this reason, different methods are being used for its removal from water and wastewaters; however, adsorption by means of geopolymers is found most suitable and effective. Alshaaer et al. [78] investigated the adsorption behavior of zeolitic tuff-metakaolin geopolymers for Cu^+2^ ions elimination and monitored the highest adsorption competence of 7.8 mg/g of adsorbent at an early zeolitic tuff:metakaolin ratio as 0.5.

### 3.5. Zinc (Zn)

Zinc (Zn) is also a metal, and its ions (Zn^+2^) are generated by industries of galvanizing, pigments, paints, etc., pollute water streams. Even if zinc (Zn) is valuable for a lot of constructive biological functions, the exposure to a higher level of Zn has been confirmed accountable for causing detrimental influences on human health in the form of muscular stiffness, gastro-intestinal distress, irritation, growth retardation, cancer, lung disorders, etc. [79]. Consequently, the treatment of both water and polluted waters has turned out to be a pressing need that must be addressed. The methods, such as absorption through cost-effective geopolymers, are becoming more and more popular because of their competence, ease and eco-friendliness.

### 3.6. Manganese (Mn)

Manganese (Mn) is a vital nutrient for both—humans and animals, however, the chronic exposure of this heavy metal to higher doses is injurious to health. It may be generated from an assortment of sources and decreasing quality. Non-biodegradable manganese can settle in living organisms, utilizing contaminated water enclosing Mn, which causes diverse diseases to take place [80]. In accordance with the World Health Organization (WHO, Geneva, Switzerland), the highest admissible Mn concentration in drinking water is considered to be 0.05 mg/L. The surplus Mn found in water is causing issues, such as Parkinson’s disease and bronchitis, as well as damaging respiratory and nervous systems. To address these predicaments, different adsorbents for Mn^+2^ ions have been examined, including volcanic ashes, fly ash, zeolite fruit nutshell, kaolinite, glycine modified chitosan, vermiculite [66,80,81,82,83,84].

### 3.7. Nickel (Ni)

Nickel is a metal with a chemical symbol of (Ni). Heavy metal nickel ions (Ni^+2^) are responsible for polluting water and wastewater and hence should be removed from them. Ge et al. [85] accounted that the metakaolin-geopolymer membrane can powerfully remove the Ni^+2^ ions from wastewater and predicted that such geopolymeric membranes can potentially find their way to be utilized for the removal of Ni^+2^, as well as other heavy metal ions from industrial wastewater.

## 4. Applications of Geopolymers for Water and Wastewater Treatment

Nowadays, geopolymers are regarded as versatile materials, which have drawn attention in scores of environmental applications, and hence, are being employed nearly in all fields of technology, for instance, in water and wastewater treatment as adsorbents or ion-exchangers, high-pressure membranes and filtration media, photo-catalysts, anti-microbial materials, pH buffers, carrier media in bioreactors and for the solidification or stabilization of water and wastewater treatment residues. The applications are not merely those; they can be employed as anti-bacterial materials following their incorporation with copper (Cu) or silver (Ag) in the course of ion exchange or supplementation of nanoparticles, namely, silver-silica nano-composites into the geopolymeric alumina-silicate matrix [86,87,88]. Thus, they are useful as anti-microbial binders with analogously modified plentiful zeolites to designate their potential usage for the disinfection of wastewaters or sub-surface water [89,90,91]. 

Since the discovery of geopolymers four decades ago, they have come quite a long way successfully [92] and gained attention, mostly due to the effortlessness for geo-synthesis with lesser emission of greenhouse gases (GHG) [93]. Their admirable properties, such as toughness, heat and fire resistance, refractory nature and radiation hardness, altogether make them promising for applications for radioactive waste containment, as well as for pozzolanic action, making them multi-functional. In realism, lots of industrial materials are derived using geopolymers. For example, fiber-based geopolymer composites are regarded as fire-resistant. Quite a lot of geopolymeric composites are positioned in metal tool coatings and the construction of cabinets of airplanes and buildings, with a view to trim down the intensity of inferno incidents [94]. The polymeric chain-like structure of geopolymers contributes higher chemical resistance, lower shrinkage and enhanced resistance to abrasion with high early mechanical strength [95]. 

At present, geopolymers are an emerging class of materials for the eco-benevolent refurbishment of decrepit infrastructures, recovery of marsh environments and sustainable reinforcement of structural amenities. The highly workable properties of geopolymers, such as water retention aptitude, when owning shear stress of ~80 Pa at a shear rate of 110 s1 and compressive strengths of ~40 MPa at 7 days curing, facilitate the tailoring of them. Furthermore, they possess attributes that make geopolymers a significant class of promising materials for the containment and capping of nuclear wastes perils [96]. Moreover, the development of geopolymer sorbents necessitates considerations for how to employ them in general practice. It is feasible to dose powdered sorbent constantly; however, then an added process phase is needed to separate the utilized sorbent, and operational costs may go higher. As a result, a choice of alternative methods is accessible to manufacture highly porous and permeable geopolymers, e.g., direct foaming, sacrificial template method, freeze casting, granulation or additive manufacturing (AM), i.e., 3D printing.

### 4.1. Geopolymers as Adsorbents or Ion-Exchangers 

The key drivers for this emerging approach for the application of geopolymers as adsorbents or ion exchangers are their outstanding mechanical and chemical stability coupled with a fairly uncomplicated, lower carbon, low-energy, user-friendly and cost-effective manufacturing process of geopolymerization [97]. There exist numerous efficient and familiar wastewater treatments to get rid of heavy metal cations present in it, including adsorption [98,99,100], ion-exchange, photo-catalytic degradation, i.e., as photo-catalysts, membrane filtration (or separation) materials, chemical precipitation, bio-remediation sedimentation, pH adjustment agents, the solidification or stabilization of water treatment residues reverse and forward osmosis [101,102,103,104,105,106,107,108,109,110,111,112,113,114,115]. Consequently, geopolymers offer viable alternatives to competing materials for water and wastewater treatment, such as conventional ceramics or synthetic zeolites, polymeric components in terms of cost, an assortment of choice, eco-impacts, and of course, performance. 

In recent times, on account of the significant attributes, such as a simple method, low-cost, environ-friendly nature, higher competence and mild micro-spheres synthesis method, geopolymers are successfully developed and commonly used as adsorbents, inorganic membranes, catalysts and for the immobilization of dangerous metal ions. Adsorption is regarded as the most efficient, uncomplicated and widespread technique for water decontamination [116,117,118,119,120]. Undeniably, activated carbon is the standard adsorbent material and is well-known as the most utilized adsorbent for water and wastewater treatment with an excellent demonstration of higher adsorption competencies for the removal of heavy metal cations, but its use gets hindered due to its comparatively high cost [121]. Hence, in spite of the exceptional adsorption ability of this material, its higher production costing is the root cause of wide-ranging research for low-cost yet effective options in recent times. Significantly, the unique porous structure of geopolymers and the existence of negative charges on the aluminum-tetrahedra of the resulting gels, they can efficiently and successfully absorb metal ions, namely, Pb^+2^, Cu^+2^, Cd^+2^, Mg^+2^, Hg^+2^, Cr^+3^, etc. [122,123,124,125,126,127].

The performance of heavy metal adsorption by geopolymers relies mostly on the structures formulated by the geopolymer gels; nevertheless, owing to the presence of numerous inert crystalline phases in them, the clear-cut correlations among the geopolymeric gels and performance of adsorption by them cannot be verified all the time. The earlier research works deal with the hydrochloric acid (HCl) dissolution to verify the degree of reaction of geopolymerization, and as inert crystalline phases are insoluble in acid, the gels and other dissolved matters can be measured based on the mass alterations. The adsorbents in powder form may necessitate the support materials use, such as porous ceramics and polymer foams, [127] to permit their industrial utilization or a separation step, such as pressure filtration after wastewater treatment course of action, both being harmful to the wastewater treatment costing besides the rising complexity of the procedures. The geopolymers can fruitfully be used as green adsorbents or ion exchangers by manufacturing them with wastes as precursors extending a systematic solution for their disposal too. Thus, the adsorption using these environ-benign adsorbents is a superb and very much progressive technique for the elimination of not merely inorganic but also organic contaminants from aqueous mediums, particularly through geopolymers, which can be produced employing solid wastes as precursors [128].

The ever-increasing diverse wastes from various fields, such as fly ash, metakaolin or low-calcium alumina-silicate solid wastes, accumulating as landfills are not only hazardous to the environmental sustainability but are also resource-wasting and polluting water, soil, etc., with lots of negative impacts on the health of living beings, and can advantageously be used as precursor resources. For instance, fly ash (FA) is being used as higher value-added applications, viz., the synthesis of FA-ZnO enclosing nanofibers for adsorption and photo-catalytic degradation of organic dyes, etc. [129]. Furthermore, the production of geopolymers enables benefiting industrial, agricultural, etc., wastes containing alumina and silica to be utilized as a resource for precursors, such as fly ash, clay, slag, raw kaolin, metakaolin and rice husks. Lots of research works used rice husk as a precursor, considering it as a silicon source useful for preparing a range of geopolymers making the costing comparatively low. Moreover, their uses for disinfection made ground, gaining the geopolymer technology substantial attention from not only researchers but also industries for concentrating on cleaner manufacturing. This is an additional advantage to offer systematic disposal of all such kinds of wastes that can be used to design a geopolymer useful for water and wastewater treatment and reclamation as well [130]. These compounds raise quite remarkable questions about their surface and structure, e.g., the almost total exclusion of diffusion of sequestered metal ions and avoidance of issues linked to leaching of geopolymer adsorbents [131]. 

As referred, geopolymers are famous for occupying inter-crossed linked bonds with cationic ends on the surface, structurally permitting the entrapment of toxic and radioactive metals through charge balancing. On the other hand, the micro-structure of geopolymer in the nano-metric scale of 5 to 10 nm frequently consists of numerous pores inside a highly porous –Al–O–Si– repetitive unit, and these voids are probable to provide room for ionic integration, replacement and balances too. Extraordinarily, geopolymers own a key structural unit analogous to zeolites; Provis et al. [132] unveiled that they possess an amorphous gel phase and nano-crystalline zeolites agglomerates. According to them, a proposal has been put forward that the geopolymers and zeolites could be inter-converted under suitable conditions. Thus, the conversion of geopolymers into zeolites is regarded as an exceptional strategy since the well-organized recycling of solid wastes from industries and the economical production of costly zeolites can be recognized concurrently. Beforehand, Cui et al. [133] authenticated using a high-resolution transmission electron microscope and particular area electron diffraction that numerous nanometer-ordered structures were detected in the amorphous geopolymer (Al_2_O_3_2SiO_2_Na_2_O7H_2_O), successfully acquiring the Na-A type molecular sieve from this geopolymer through a hydrothermal reaction. Afterward, a series of sodium (Na)-zeolites, including sodium (Na)-A, faujasite, sodium (Na)–P and sodium (Na)-X were attained via the hydrothermal alteration of geopolymers when sodium cations (Na^+^) were present, which displayed huge potential for membrane separation and bulky adsorbent utilizations. It is eminent that the development and nucleation of zeolites rely upon the concentration of alkali metal cations and their kind [23,134,135,136,137]. 

The zeolites, which are derived from geopolymers, are acquired from a solution of Na^+^ cations resulting in restraining their diversity. Hence, it is imperative to develop an extensive variety of zeolites from geopolymers for utilizations linking bulky adsorption and membrane separation. On the other hand, in a research study, Rossi blended a biomass fly ash as 75% with 25% geological natural metakaolin and used the mixture as a precursor for geo-synthesis through geopolymerization and achieved an end-product with a huge specific surface area of 56.35 m^2^/g together with a brilliant pressure resistance of up to ~10 MPa. Furthermore, a noteworthy improvement in adsorption competence and compressive strength was monitored in a study whereby the supplement of cork waste residue was made into metakaolin-based geopolymers [138]. Moreover, the existence of dyes in wastewaters is considered a somber crisis for ecology [139]. Cation-enclosing dyes, such as methylene blue or methylthioninium chloride with a formula C_16_H_18_ClN_3_S, is a salt that is cationic and used as a dye, mostly in the textile industry for cotton, wool and leather dyeing, paper and plastics, as well as furniture coloring. Methylene blue is very injurious to the health of living beings since it can cause blindness, allergy, abdominal disorders, asthma, etc. [110,139]. As a result, such dyes must be removed from aqueous solutions by means of ultra-filtration, photo-degradation or ion-exchange methods. Still, adsorption is one of the most efficient and affordable routes for water and wastewater treatments [2,140]. For the reason that it is a cationic dye, hence, positively charged; the application of geopolymers with negatively charged networks as adsorbents is the most promising solution in this context. Furthermore, the likelihood of the development of zeolites in the course of the geopolymerization of metakaolin can optimistically influence the procedures of adsorption. 

The zeolite is portrayed by a higher selectivity headed for methylene blue [141]. The few modern research works are throwing light on the application of zeolite-type geopolymeric green nano-adsorbents considering their higher effectiveness, steadiness, trouble-free production system, exceptional binding characteristic and attractive lower costing [99,141,142,143,144]. They are found highly porous and can be manufactured at as low as room temperature through a simple process of geopolymerization among aluminum and silicon sources in an alkali medium [99,143]. Zeolites resembling geopolymers are appreciably useful for a variety of eco-friendly applications, viz., the taking away of heavy metals and photo-catalytic degradation of a few organic compounds, such as benzophenone, metronidazole, p-nitrophenol and eriochrome black T [145,146,147,148,149,150], along with some significant industrial uses, namely, diversion of methanol (CH_3_OH) to olefins and phenol hydroxylation for water and wastewater treatment.

### 4.2. Thermo-Dynamics of Adsorption 

The necessary data with regard to the nature and thermo-dynamic viability of the adsorption progression can be obtained with the help of the thermo-dynamic parameters, standard change of entropy (DS0), change of standard free energy (DG0) and change of standard enthalpy (DH0). In the context of geopolymer adsorbents, the accounted DHO values of adsorption are commonly found as positive [27,102,113], designating with respect to the adsorption competence that it escalates at elevated temperatures. Nevertheless, one differing upshot as DH0 10 kJ/mol was found reported by Li et al. [151] in the case of elimination of methylene by fly ash-based geopolymer. This is additionally supported by the fact that the values of DS0 are constantly positive, signifying that the system entropy enhances are subsequent to the adsorption course of action [113,151,152]. This can be comprehended with the separation of hydrated water molecules from the metal ion, i.e., inner-sphere complexes are formulated, and specific adsorption takes place previous to the attachment to the surface sites of geopolymers, which is regarded as an energy-requiring procedure [27,113,152].

### 4.3. Adsorption of Radioisotopes

Fantastically, geopolymer composites are employed to adsorb a number of elements that have radioisotopes, viz., caesium (Cs), strontium (Sr), radium (Ra) and cobalt (Co). The elimination of radioisotopes from water has turned out to be progressively more significant in the aftermath of the incident of the accident at Fukushima nuclear site, Japan, in 2011 [153]. In the aqueous environment, caesium, which is highly soluble and stable, occurs as Cs. The radioactive isotope 137Cs is of great concern because it has a long half-life of 30.2 years. On the other hand, strontium is found present chiefly as Sr^+2^ in the aqueous environment and possesses quite a lot of radioactive isotopes. Of which, 90Sr with a half-life of 28.9 years is the most noteworthy [154]. Usually, all radium (Ra) isotopes are found present as Ra^+2^ in low salinity conditions with half-lives ranging between a few days and 1600 years. The metakaolin-based geopolymer is potentially effective for the adsorption of the caesium (Cs), and therefore, preferable to lead (Pb^+2^), copper (Cu^+2^), cadmium (Cd^+2^), nickel (Ni^+2^) and zinc (Zn^+2^). The adsorption competence is not influenced by the presence of sodium chloride (NaCl) up to 10 weight-% concentration. Chuang and Liao [155] adsorbed caesium (Cs^+^) ions by phosphate-based geopolymer enclosing potassium (K^+^), zinc (Zn^+2^) cations and Ferrocyanide anion [(Fe(CN)_6_)]^−4^. Lee et al. [156] utilized porous geopolymeric blocks manufactured with fly ash and blast furnace slag with a view for eliminating Cs^+^. Geopolymer composites are compared satisfactorily with so many other types of adsorbents for the removal of Cs^+^, which show the highest adsorption competence [153]. Chen et al. [157] investigated the elimination of Cs^+^, Sr^+2^ and Co^+2^ ions by metakaolin and fly ash-based geopolymer composites and achieved very high adsorption competencies. Moreover, it is possible to adsorb these ions with 0.1 M HCl, which suggests that the adsorbent could be regenerated [157]. Geopolymer composites using fly ash are reported to be potentially efficient for taking away cobalt (Co^+2^). At this juncture, it is interesting to note that both the radioisotopes and non-radioactive isotopes exhibit analogous aqueous chemistry. In the end, radium isotopes can be significantly removed by foamed geopolymers where there is an incorporation of barium sulfate (BaSO_4_), and the material is suggested to be employed as a passive filtration material. Thus, geopolymer composites can brilliantly remove different polluting ions efficiently. 

### 4.4. Adsorption of Heavy Metal Ions 

Regrettably, the perilous and lethal wastewater carrying with it a range of heavy metal ions has turned out to be a universal environmental apprehension attributing to not merely so many human and animal diseases through contaminated water but also fueling the issue of the freshwater scarcity on our planet [158,159,160,161]. The degradation of an organic molecule and heavy metal confiscation from wastewater is a chief topic of public attention because of its relevance to the environment and human health. Thus, the heavy metal contamination in wastewater is exceedingly creating a negative impact on the environment due to their toxicity and its direct drain in natural surface water bodies [39]. For these reasons, plenty of types of materials are being used as adsorbents to solve the purpose of removing heavy metals from water and wastewater; however, the complexity of their synthesis and the higher costing of their precursors has been compelled to limit their widespread use. Therefore, geopolymers are being used as an adsorbent for the removal of ions of different heavy metals, namely, chromium (Cr), calcium (Ca), cesium or caesium (Cs), magnesium (Mg), arsenic (As), nickel (Ni^+2^), zinc (Zn), copper (Cu^+2^), cadmium (Cd^+2^), lead (Pb^+2^), NH^+4^, manganese (Mn^+2^) and Co^+2^, as well as methyl violet and methylene blue along with anions—phosphate, fluoride, and radionuclide of 137Cs and 90Sr, plus dyes, etc. [162,163,164,165,166,167,168,169,170,171]—from water and industrial wastewaters, which were found superbly suitable and valuable for adsorption, proving to be the most extensive and useful method.

Just recently, some endeavors to utilize both fly ash and metakaolin-based geopolymers as adsorbents for heavy metal ions and dye removal from wastewaters were undertaken. The fly ash-based geopolymers are employed to eliminate Cu^+2^ ions and Pb^+2^ with significant success [26,171]. In contrast, Cheng et al. [172] investigated a metakaolin-based geopolymer playing the role of an adsorbent for removing Pb^+2^, Cu^+2^, Cr^+2^ and Cd^+2^ from water and monitored the efficacy for Pb^+2^ subtraction in particular. López et al. [170] optimized the chemistry of geopolymers keeping the molar ratio of Si:Al as 2.0 for the choosy adsorption of Cs^+^ and Pb^+2^ from the solution mix of other heavy metal ions. Tang et al. [173] studied the fabricated porous metakaolin-geopolymer spheres having 54 m^2^/g surface area, 15 nm pore size and 60% porosity to take out Cu^+2^, Pb^+2^, Ca^+2^ and other polluting ions from wastewater and projected that the porous geopolymer spheres are cost-effective, expedient and environ-benevolent adsorbent for metal ion removal. 

On the other hand, Andrejkovicova et al. [174] examined the high performance of metal removal for the metakaolin-based geopolymer mix and clinoptilolite, which was used as a filler and accounted for the adsorption of metal cations on geopolymers that is found to be well fitted by the Langmuir model and 25% clinoptilolite adding on provided the optimum upshots for the subtraction of Pb^+2^, Cd^+2^ and Zn^+2^. Furthermore, Barbosa [163] made a comparison of methyl violet 10B dye adsorption behavior of the metakaolin-geopolymer and its permeable counterpart and found that the adsorption competence of the geopolymer was enhanced in porosity, with the greatest adsorption capability of mesoporous geopolymer reported as 276.9 mg/g. 

Geopolymeric adsorbents can be employed as powders, granules and monoliths too; however, in the case of packed beds, the powdered adsorbents cannot be used in contrast to monoliths [167]. The remediation of contaminated water is of prime significance because of the escalating freshwater consumption of water, resulting in a grave shortage in most large parts of the world. To address this challenge, water and wastewater treatments, by means of geopolymers, have proven to be unique owing to their three-dimensional (3D) network structure, which marvelously makes geopolymers the most promising adsorbents [175,176]. Previous studies have revealed the immobilization of different heavy metals in geopolymers, for instance, Pb^+2^, and shown that geopolymers are competent enough to absorb the heavy metals Pb^+2^, Cu^+2^, Cr^+3^, Cd^+2^, etc. [172,177]. The synthesis of a kaolin-based geopolymer was performed by Naghsh and Shams [126] in interest to review adsorption competence for Ca^+2^ and Mg^+2^, and utilized “cetyl trimethylammonium bromide” to modify geopolymers and to enhance the adsorption ability for Cu^+2^. 

Noteworthily, Bentonite—a naturally accessible low-cost clay—possesses one-of-a-kind attributes, namely, high surface area, higher alumina/silica ratio, high amorphous content, chemical stability, elevated porosity and far above the ground capability of exchange of cations, making it valuable for dissimilar utilizations for geopolymers [178,179]. Both the perilous organic molecules and heavy metals are identified as noxious originators for the sickness of the common people [180]. First and foremost, they are discharged into the atmosphere through the actions of humans and/or industry [181]. For this reason, research on multi-functional materials that deal with concurrent energy manufacture and environmental remediation is related to modern-day technologies associated with cleaner production. 

Presently, apart from geopolymer composite-based adsorbents, a range of other adsorbents are rising; however, most of them are found in powder form [182,183]. It is tricky to recover and regenerate these adsorbents, which may cause secondary heavy metal sludge contamination and is not conducive to constant function. When the powder is much finer, then it floats on the wastewater and is uncomfortable to recycle, and the pressure passed through the column is found to be much higher. The outsized particles are simple to post-process; however, the pressure passed through the column is much smaller, and hence, it may diminish the adsorption competence. The micro-spheric adsorbents are regarded as one of the most widespread adsorbents employed in the column for dynamic removal since they possess a fairly huge diffusion resistance and are trouble-free to disassemble and separate, as well as the pressure through the column being opposite [59].

### 4.5. Dyes

The subtraction of dyes from industrial wastewaters is of paramount significance, not merely because most of them are toxic and demonstrate carcinogenic attributes, but also for the reason that treated industrial wastewaters can play a role of an imperative source of clean water to provide relief to the most pressing need of freshwater demand from the society. The existence of trace quantities of dyes for <1 ppm in industrial wastewaters is tremendously evident, which is redundant [184]. One of the most universally employed dyes is methylene blue, a cationic dye, which is proven to cause blindness, abdominal disorders and respiratory distress [110,184]. The dyes and their breakdown yields are also venomous, mutagenic and carcinogenic and cause irritation of eyes, sore throat, skin, asthma and allergic contact dermatitis on exposure to organic dyes [185,186]. That is why its subtraction from wastewaters is highly obligatory. Quite a lot of techniques, such as photo-degradation, ion exchange and ultrafiltration, are utilized for methylene blue elimination; however, adsorption is still regarded as one of the most successful, simple and low-priced methods. Even though activated carbon represents elevated adsorption competence, its extensive application is confined due to the higher manufacturing cost [110,184,185,186,187]. Consequently, new-fangled and cost-effective options are practiced. One such alternative of geopolymers is emerging swiftly since they are inherently porous, having a negatively-charged alumina-silicate network balanced by cations of Na^+^, K^+^, etc., symptomatic of the practicability of being applied as adsorbents [188,189,190,191,192]. There are reports on their application as powders for dye removal and as granules, as well as monoliths for heavy metal pulling out from wastewaters [26,188]. The synthesis of fly ash-ZnO containing nanofibers is used not only for the adsorption but also for the photo-catalytic degradation of organic dyes. On the other hand, one significant cationic dye of methylene blue present in wastewaters can be absorbed by a number of mechanisms, such as electrostatic or ionic interactions, active binding by oxygen groups, photo-degradation and π–π conjugation [189]. One more imperative occurrence is the “adsorbate transport” from the bulk solution to the sorbent surface, which realizes in quite a lot of steps. Generally, the course of action of adsorption can be controlled by only one step, e.g., film or external diffusion, surface diffusion, pore diffusion and adsorption on the surface of the pore, or the grouping of more than one step [2,190]. However, the application of porous geopolymer monoliths for methylene blue (MB) extraction from wastewaters was also accounted for [2,190]. The MB elimination capability of the monolithic bodies figured 15.4 mg/g, while the adsorbent can be reused up to five times. Thus, the hopeful outcomes exhibited the practicability of the use of porous bodies, and not powders, to pull out MB from contaminated wastewater. Nonetheless, the exclusive competence of adsorbents is trimmed down considerably, to about 65%, when the MB early concentration touched 50 ppm. Consequently, more investigations for addressing the employment of bulk porous geopolymers should throw light on the most dominant parameters influencing the adsorption of dyes by the geopolymers. One option to improve the MB adsorption capacity of geopolymers, compared to the utilization of cylindrical discs with a thickness of ¼ 3 mm and d ¼ 22 mm, could be the application of porous geopolymer spheres (GS) with a size of 2 to 3 mm [2].

The adsorption of dyes using geopolymer composites as adsorbents is a significant topic, and hence, must be understood thoroughly. Quite a lot of geopolymers have been applied so far for the subtraction of the basic cationic dyes, such as methylene blue and crystal violet. Yousef et al. [191] prepared an effectual methylene blue adsorbent with a Langmuir capacity of 4.75 mg/g using sodium hydroxide (NaOH) for activating kaolin and employing zeolite as a filler. Furthermore, Li et al. [151] manufactured a fly ash-based geopolymer by means of a solid-state fusion technique with sodium hydroxide (NaOH) and obtained higher capacities, to some extent, of 18.3 and 17.2 mg/g for methylene blue and crystal violet, correspondingly. With a view of absorbing methylene blue, a competent geopolymer was manufactured, and it obtained an adsorption capacity of 50.7 mg/g experimentally. Furthermore, their adsorbent demonstrated enhanced porosity, and it is found floatable because of hydrogen peroxide (H_2_O_2_) supplementation. Geopolymers are exhibiting promising characteristics for the elimination of dye, namely, the aptitude to be regenerated [184]. Barbosa et al. [163] manufactured a geopolymer based on metakaolin, rice husk ash and soybean oil as a mesostructured directing agent and employed it for the adsorption of crystal violet and achieved a comparatively higher maximum capacity of 276.9 mg/g.

### 4.6. Adsorption of Ammonium, Sulfate, Fecal Coliforms and Phosphorous

Geopolymer composites are valuable for the adsorption of ammonium ions (NH_4_^+^), sulfate, fecal coliforms and phosphorous (P). Attractively, one precise, unique and interesting application of geopolymers is for the subtraction of ammonium (NH_4_^+^) ions from wastewater [162,192]. Ammonium (NH_4_^+^) ions are generally found in nitrogen species, such as untreated wastewater. The elimination of NH_4_^+^ ions from wastewater is investigated expansively by means of ion exchange on natural and synthetic zeolites as an option to the customarily employed microbial nitrification–denitrification [193,194,195,196]. With a view to prevent “eutrophication”, the legislative requisites for removing nitrogen (N) from municipal and industrial wastewaters turn out to be progressively more common in the present time. Indeed, ammonium (NH^+4^) is the nitrogen species that contributes the highest to the “eutrophication” of water bodies, whereby nitrogen (N) is the nutrient in poor supply [197].

The anaerobic digestion followed by natural zeolite-based ion exchange is potentially applicable for the recovery of nitrogen with a low operational cost and superior performance for nitrogen removal when compared to the traditional Anammox procedures or nitrification–denitrification [198]. The conventional methods to get rid of the nitrogen (N) are found based on the microbial nitrification–denitrification processes and technologies, which may become ineffectual at lower temperatures with high operational costing when compared to the process based on ion-exchanging [199]. Marvelously, the geopolymers have a very high affinity for NH_4_^+^ for the charge-balancing Na^+^ cations in the alumina-silicate network of geopolymers, which may be exchanged to NH_4_^+^ at nearly 100% competence [200], provided that the charge-bearing sites are within reach. The ion exchange aptitudes of powdered metakaolin geopolymer for NH_4_^+^ is accounted as 18 to 32 mg/g [162], relying upon the examined water matrix, i.e., raw wastewater subsequent to the screening or following the advanced primary treatment, which is analogous to a lot of synthetic or natural zeolites. Furthermore, the granules of the geopolymer are investigated for ammonium (NH_4_^+^) exclusion on a bench-scale column experiment occurring in a plant for wastewater treatment: < 4 mg/L NH_4_^+^ is constantly reached at a water temperature of 10 °C; generally, 12 °C is the maximum limit for a well-organized operation of nitrification–denitrification processes, and the material can be reproduced with chloride of sodium (NaCl)/sodium hydroxide (NaOH) [192]. Appealingly, the elimination of ammonium (NH_4_^+^) ions from wastewater by means of exchange with sodium (Na^+^) ions is one precise application of geopolymer sorbents prepared through three-dimensional (3D) printing, i.e., additive manufacturing [162]. Moreover, they have been examined as “adsorbent” or “ion exchanger” [27,102,103,105,110,164] materials for membrane agents for pH adjustment photo-catalyst, for solidification or stabilization of water treatment residues in water and wastewater treatment. 

Geopolymers have a higher affinity for ammonium (NH_4_^+^) ions, the charge-balancing Na^+^ cations in the geopolymer alumina-silicate network and may be exchanged with NH_4_^+^ ions up to nearly 100% competence provided the charge-bearing sites are available. Luukkonen et al. [162,165,192] utilized a metakaolin-based geopolymer composite and achieved 21.07 mg/g ammonium (NH_4_^+^) adsorption capacity in model solutions, which is greater than using typical natural zeolites. Furthermore, they reported that the elimination was based on ion exchange, and the material can be regenerated with a solution having 0.2 M sodium chloride (NaCl) and 0.1 M sodium hydroxide (NaOH), and the adsorbent was found effective for site-treatment when landfills initially leachate NH_4_^+^ at 55 mg/L at lower temperatures [165]. 

In one more research study by Luukkonen et al. [162], where they employed a central composite design method to optimize the metakaolin-based geopolymer composite manufacturing in order to maximize ammonium (NH_4_^+^) adsorption capacity. When higher quantities of silicate and hydroxide are employed, a low content of metakaolin and sodium (Na^+^) ions as a charge-balancing cation in spite of potassium during the production resulted in the highest capacity. This type of optimization has likely augmented the highest adsorption capacity from 21.07 to 31.70 mg/g. Luukkonen et al. [192] also investigated a metakaolin geopolymer produced with the granulation-geopolymerization technique for employing it as a filter media. It is probable to achieve constantly less than 4 mg/L ammonium ion (NH_4_^+^) concentration in wastewater from municipal drainage with initial NH_4_^+^ and 23 mg/L at a lower temperature of more or less 10 °C and regenerate material multiple times with sodium chloride (NaCl)/sodium hydroxide (NaOH). Bai and Colombo [201] investigated metakaolin-based geopolymeric foams as monolithic porous filters manufactured by means of 3D printing, utilizing the direct ink writing approach. The filter is found capable of taking away up to 95.3% of the ammonium (NH_4_^+^) from the initial concentration of 3 mg/L of NH_4_^+^.

On the other hand, sulfate (SO_4_^−2^) is a naturally-occurring ever-present anion that is not regarded as toxic, even though it can be responsible for the salinization of surface water bodies. For that reason, the elimination of sulfate (SO_4_^−2^) should be managed in numerous industries of mining, such as drainage from acid mine and plants of desalination, wherein reverse osmosis reject water. Runtti et al. [113] tailored a blast furnace slag-based geopolymer through ion-exchanging charge-balancing sodium (Na^+^) cations into barium and utilized the achieved material to get rid of sulfate from synthetic wastewater with (SO_4_^−2^) at 1000 mg/L, as well as mine effluents with (SO_4_^−2^) at 900 mg/L. The monitored adsorption capacity was comparatively higher, at around 119 mg/g, and it was likely to obtain very low sulfate concentrations of 2 mg/L. The projected removal mechanism was the precipitation of the tremendously lower solubility of barium sulfate (BaSO_4_) or surface complexation [113]. Zhang et al. [202] displayed, by molecular dynamics simulation, that cations, viz., sodium (Na^+^) or magnesium (Mg^+2^), are adsorbed on the surface hydroxyls of the N–A–S–H gel and attract the ions of (SO_4_^−2^).

### 4.7. Surfactants 

The name “surfactant” is derived from the mixture of words surface-active agent, meaning “active agents on the surface”. Such compounds trim down the surface or interfacial tension among two fluids, or one fluid and gas, or a liquid and a solid active as wetting and foaming agents, detergents, dispersants and emulsifiers. They are one of the hottest up-and-coming contaminants contributing to boosting the pollution of global water resources. Basically, they are organic surface-active agents employed for a range of applications in the field of polymers, fibers, metals, paints, pharmaceuticals, paper and pulp, micro-electronics, cosmetics, foods, etc. [203]. The category of surfactants is being confirmed on the data of their charge on the head group. A total of three classes of surfactants, viz., non-ionic, ionic( anionic and cationic) and amphoteric (enclosing both the types of charges), are found. More or less 60% of the surfactants developed are from the anionic class, and roughly 12 M tons of surfactants are utilized internationally [204,205,206]. 

Globally, the consumption of an elevated quantity of anionic surfactants is assigned to their simple synthesis and good-quality cleaning, as well as the foaming attributes [207]. Alas, the negatively-charged surfactants are accountable for detrimental impacts to not only humans but also fish, vegetation, etc. For instance, they can be the reason for the eutrophication of effluent treatment plants and produce foam in freshwater-bodies on the earth. Notably, the concentration of surfactants was found in ranges from 1 to 10 mg/L in domestic wastewater, while in industrial wastewater, it reaches the level of 300 mg/L [208]. It is quite noteworthy at this stage that the standards of the World DOE permit for merely 1.0 mg/L of anionic surfactants in surface water bodies. Consequently, it is very much essential to get rid of these most modern pollutants in the form of anionic surfactants from industrial effluents before their discharge into freshwater resources on account of their higher level of usage, concentration limits, as well as negative impacts on environments and health of living beings. With a view to remove anionic surfactants from the wastewater, loads of techniques for its treatment are being used presently, such as chemical and electrochemical oxidation, ion-exchange and membrane separation, microbial treatment, foam separation, coagulation and some other special adsorption techniques [209,210,211]. However, a few of the mentioned techniques are restricted on the basis of efficacy, costing and eco-benevolence. Appreciably, adsorption is the leading and fitting process for eliminating surfactants owing to its practicability, cost and course convenience. Scores of adsorbents, namely, multi-walled carbon nanotubes (MWCNTs), geopolymers, activated carbon, chitosan, graphene, coal, zeolites and fly ash, are being used for the purpose of surfactants subtraction from wastewater [212,213,214,215,216]. Additionally, MWCNTs have a very interesting evolution of the dielectric characteristics in frequency and temperature variation due to the polarization mechanisms present. At frequencies in the MHz order, it appears that the polarization of orientation increases with the rise of temperature. The electrical conduction of this material increases with increasing frequency. If at low frequency this material has an insulating character, with increasing the frequency, it has a semi-conductor character [217]. 

Among all these, the fly ash-based geopolymer exhibited first-rate performance to address the removal predicament of an anionic surfactant. The surface area can be improved by changing the geopolymer chemistry. When surfactants are being employed, they stabilize the formulated foam, and the resultant materials contain extremely interconnected pores and permeability akin to granular filters [218]. Furthermore, they can be prepared on-site by supplementing vegetable oils, i.e., triacylglycerols, to the alkaline geopolymer suspension resulting in the saponification reaction, meaning decomposition into glycerol and soaps due to higher pH [219]. Significantly, the albumin powder obtained from the chicken egg is examined for use as a surfactant for foaming geopolymers with hydrogen peroxide (H_2_O_2_) [201].

## 5. Geopolymers as Membranes and Filters

The application of monolithic bodies might permit the direct use of geopolymers in packed beds as membranes that would appreciably make the procedure simpler. The production of fly ash-based geopolymeric micro-filtration membranes has drawn increasing interest [220]. Furthermore, zeolites are being classed as inorganic alumina-silicates, which typically enclose a number of channels smaller than 2 nm in size and zeolite membranes. These materials are receiving escalating interest for an assortment of applications, such as catalyst reactors and flow batteries [221]. Among the present accessible methods of eliminating heavy metals, membrane separation offers a distinctive benefit by treating the ions irrespective of their concentrations [222]. Specifically, the membrane can improve trace ions in a solution to a comparatively higher concentration for analytical chemistry utilizations [223,224], whereas for the ions with higher concentrations, the membrane can additionally enhance them for recovery purposes [225]. In reality, before the flame atomic absorption spectrometric determinations, several investigations employed membranes for the pre-concentration of trace ions in environmental specimens. A lot of industrial areas brought them into play for practical utilization for their accurateness and operability. Although studies on the enhancement of ions with comparatively higher concentrations for advanced higher value-added application are somewhat inadequate, as such investigations have a key focus on immediately sought-after ions, such as lithium (Li) and magnesium (Mg) [226]. Even though a few reports emphasize the recovery of membrane-separated heavy metal ions; they are focusing on ion concentration effectiveness. Conversely, some of the examinations have concentrated on the on-site application of heavy metal ions attached to the membrane [227,228,229,230]. Even so, loads of investigations have authenticated that geopolymers with concentrated mesoporous or macroporous structures can productively be produced, and a few researchers tested their membrane separation performance [85]. What is more, geopolymers show evidence of superior thermo-stability and resistance to corrosion that is expedient for advanced alteration subsequent to the separation when compared to conventional organic membranes [39]. 

### 5.1. Pressure-Driven Membranes

The pressure-driven membrane course of action is employed to either refine or concentrate the weak solutions or dispersals where the size of molecules or separated particles, as well as chemical attributes of the solvent, are crucial in making the choice for a suitable membrane. On the basis of the separation capacity, the pressure-driven membranes are categorized as micro-filtration, ultra-filtration, nano-filtration and reverse osmosis. Among these types, the geopolymeric membranes are characteristically found in the range of micro or ultra-filtration with pore sizes between 20 and 100 nm [85]. The intrinsic porosity of the geopolymer composites is valuable for membrane utilization. It means that its making procedures do not fundamentally necessitate any precise additives or foaming procedures. 

Nevertheless, one of the early geopolymer membranes was made by employing the sacrificial filler technique, wherein the self-assembled strata from nano-size polystyrene spheres are prepared and then covered with a slurry made up of a mix of metakaolin/alkali silicate, after that, 200 MPa pressure is applied for 1 h and curing is executed at 50 °C for one full day, i.e., 24 h, and the polystyrene template is dissolved into a solvent [231]. Furthermore, Ge et al. [85] manufactured a geopolymeric membrane straightforwardly, blending the metakaolin with sodium silicate (Na_2_SiO_3_) and curing at 60 °C, whereby the total porosity membrane possessed was 62.64%, and water flux values at steady-state were roughly ranging between 21 and 237 kg/m^2^ (9 h) at 0.1 MPa, relying upon the ratio of H_2_O/Na_2_O. What is more, the membrane can absorb Ni^+2^ ions with a capacity of 43.36 mg/g [85]. Xu et al. [111] prepared a similar membrane using metakaolin with an optimum ratio of H_2_O/Na_2_O as 18 with a water flux of 185 kg/m^2^ (9 h) at 0.3 MPa when the membrane thickness was 5.0 mm, and there was a 100% rejection rate for nano-sized Al_2_O_3_-particles. Additionally, the geopolymer membranes having the potential to eliminate Ca^+2^ and Mg^+2^ are also found reported [232]. 

Geopolymer membranes based on blast furnace slag were manufactured by the hydraulic pressing technique wherein the optimum conditions for manufacturing were 400 bar pressure, 6 h drying time and 5 min pressing time, as well as 225 bar pressure, 8 h drying time and 30 min pressing time for chemical oxygen demand (COD) removal as 100% and permeation flux optimization at 1960 kg/m^2^ (9 h), correspondingly [233,234]. One more technique for the application of geopolymer technology for membrane manufacturing is to first prepare an amorphous geopolymer material and alter it into crystalline zeolite membrane by means of a hydrothermal aging procedure, e.g., self-supporting membranes of Na-A zeolite manufactured with geopolymeric material by immersing them into water or a dilute solution of sodium hydroxide (NaOH) at 90 °C for 6 to 48 h [234]. Moreover, if the ratio of sodium (Na)/silica (Si) is boosted up to 1.25, the conversion from amorphous geopolymer to zeolite can take place through a plain heat curing at 60 °C devoid of hydrothermal treatment [234]. Furthermore, geopolymers can be cured as the faujasite—a type of zeolite—by alternatively supplementing oleic acid (C_18_H_34_O_2_) and powder of aluminum (Al) to endorse the permeability [235]. Though the referred membranes are utilized for the water/ethanol separation, it is found displayed that the zeolite membranes also demonstrated the carry potential in water treatment utilizations [236].

### 5.2. Geopolymeric View on Filtration Media

Magnificently, geopolymer composites are not only valuable for the high pressure-driven membrane application but also useful for filtration media in sand filters, point-of-use water, permeable reactive barriers, treatment filters, etc. This simply means that besides simple physical filtration, geopolymer materials are competent enough to serve the purpose as catalytically-active media and/or as adsorptive. Their use as filtration media essentially necessitates pores in the range of micro or millimeters besides micro and mesoporosity with a view to possess adequately higher permeability. Their production techniques of exceedingly porous geopolymers categorized as direct foaming, replica method, additive manufacturing or 3D printing, sacrificial filler method and a few others, such as granulation, are also considered [97]. Among these, direct foaming is the most extensively employed method, which includes mechanical blending or blowing of agents to induce gas bubbles into the fresh geopolymeric paste [97]. 

Estimably, geopolymer foam is found competent enough to absorb 95% and 87% of ammonium (NH^+^) and copper (Cu^+2^) ions, in that order, from synthetic wastewater at the time when the initial concentration was kept as 3 mg/L for each of them [201]. The total and open porosities of these geopolymer foams are reported ranging from 67 to 88 and 60 to 85 vol%, correspondingly [201]. Landi et al. [237] manufactured geopolymeric filter material using metakaolin, potassium silicate (K_2_ SiO_3_) and fumed silica with exceedingly interconnected macro-pores by means of elemental silicon as a blowing agent. The filter possessed NH_4_^+^ ion exchange capability, though it is concluded that merely a smaller segment of the internal volume pores was found accessible because of the cavity dimensions. Pervious geopolymer concrete made up of fly ash, nano-silica and ordinary Portland cement (OPC) could be employed for the elimination of fecal coliform and phosphorus from wastewater on account of a boost in pH and leaching of calcium, correspondingly [238]. The cautiously controlled size, shape and amounts of pores of geopolymeric materials can be developed through a novel technique of 3D printing or additive manufacturing [97]. 

The geopolymers are investigated as catalysts or catalyst supports for the degradation of a contaminant in both liquid and gaseous phase reactions [239]. Thus far, geopolymers are applied as photo-catalysts for the degradation of an assortment of dyes in the treatment process of water and wastewater. In this case, the reactive radicals are produced when the catalyst undergoes irradiation with the equivalent or greater energy than the bandgap. Furthermore, geopolymer catalysts can be utilized in some other sorts of advanced oxidation course of actions and for other recalcitrant contaminants of organic origin; however, such investigations are still undersupplied. Captivatingly, geopolymers are attention-grabbing materials as catalysts since they can be designed to have higher permeability, durable chemistry, surface area and, of course, accepted mechanical strength along with an uncomplicated, economical and reduced operational energy synthesis with a low carbon footprint. For the most part, the method of production of adsorbents or porous materials is being followed for the manufacturing of geopolymeric catalysts and catalyst supports, for instance, the mesoporous microstructure of geopolymer catalysts.

The metals that are active catalytically can be brought into the structure through blending them into the early-state geopolymer paste as salts or nanoparticles, or to the cured material using direct ion exchange or through firstly converting the geopolymer into ammonium (NH_4_^+^). The benefit of carrying out the Na^+^ to NH_4_^+^ ion exchange stage first is that the succeeding exchange of ions by means of catalytically-active metal is more competent [240]. What is more, the modus operandi of wet and incipient wetness impregnation has been brought into play for the supplement of catalytically-active components to geopolymers. Additionally, a few raw materials from wastes already enclose catalytically-active metals with them, as found reported in a study by Zhang and Liu [241], whereby fly ash was a precursor for the production of geopolymers. Here, fly ash is found to contain an adequate quantity of oxide of metal semi-conductors, Fe_2_O_3_ as 4.78 weight% and TiO_2_ as 0.94 weight%, to contribute to photo-catalytic activity to degrade the dye. The cadmium (Cd^+2^) suspension-geopolymer spheres (CdS-GS) are used for photo-catalytic degradation of methyl orange by the suspension–solidification technique along with the photochemical synthesis practice wherein the CdS crystals development is encouraged by UV-irradiation [115]. The solutions with 5 mg/L concentration of methyl orange are constantly treated with 0.4 mL/minute at 25 °C in a quartz tube reactor packed with catalysts of CdS-GS under ultra-violet light. The methyl orange could be eliminated up to a total of 93%, i.e., 39% adsorption plus 54% photo-degradation, by means of a bed of 5 g of CdS-GS and a 2 h contact time. Likewise, the resistance against catalyst poisoning is examined via the repetition of five cycles: the methyl orange abatement was still beyond 90%, signifying that the CdS geopolymer-spheres had a comparatively stable photo-catalytic recital. The methylene blue, another dye, is photo-catalytically degraded by making the use of geopolymers based on fly ash or metakaolin [115,241]. 

Incorporation of the active component, TiO_2_, with the metakaolin-based geopolymers by way of ion exchange is performed by Gasca-Tirado et al. [240]. The geopolymers treated through the ion exchange technique have exhibited larger surface areas than those with no active component. The greatest Ti-content is obtained with the geopolymer cured at 90 °C. Following 90 min of reaction, the color of the methylene blue was found to have vanished absolutely. Zhang and Liu [241] brought into play the fly ash-based geopolymeric photo-catalyst with no adding up of active metals using a contact time of six hours and successfully achieved a 93% abatement of methylene blue. Nevertheless, the adsorption was found accountable for 89% of the methylene blue subtraction. Fallah et al. [242] reported from their study that the majority of the methylene blue degradation is also on the grounds of adsorption of the dye onto the Cu_2_O nanoparticle-blended metakaolin-based geopolymer with an inconsequential mechanism of UV-induced degradation. 

Zhang et al. [243] manufactured a geopolymer composite employing bottom ash and graphene enclosing manganese (Mn^+2^) ions and copper oxide (CuO) as active metals. The metals are induced through conducting the first ion exchange of Na^+^ to NH_4_^+^ and immersing the material into the solutions of Cu(NO_3_)_2_.3H_2_O and Mn(NO_3_)_2_. The calcination at 400 °C for four hours is carried out to alter copper into oxide. The catalyst is examined for hydrogen (H_2_) gas production, as well as the photo-catalytic degradation of aqueous direct sky blue 5B dye fruitfully.

### 5.3. Geopolymers as a pH Adjustment Agent 

In the course of wastewater sludge bio-gasification, a significant plunge in pH can take place owing to the development of the acid through bacteria metabolism, posing a challenge for the upholding of a constant pH. It is known that the geopolymers are potential pH-buffering materials since they enclose free leachable alkalis in the pore solution [1,106,109,167,244]. Moreover, the fixed-film wastewater treatment courses of actions using bio-film reactors employ diverse kinds of floating carrier media to facilitate the adhesion and biofilm development, e.g., the carrier materials comprising polystyrene and light-weight expanded clay aggregate are already utilized. Silva et al. [245] manufactured geopolymeric bio-film carrier media of size 2–3 cm from thermally-treated tungsten-containing mine waste mud, sodium silicate (Na_2_SiO_3_) and sodium hydroxide (NaOH) at 800 °C, for 2 h. One of the mixtures exhibited good-quality potential in the context of pH, which should be inferior to 8 and should have stability when immersed in water. For this reason, the geopolymer-based bio-film carrier media is an economical option for wastewater treatment work.

## 6. Geopolymer Interaction Mechanism with Heavy Metals

Geopolymer removes heavy metals via an adsorption process in which heavy metal ions, or adsorbates, cling to the accessible binding surfaces of geopolymer. The adsorption isotherm is required to explore the nature of the interaction between geopolymer and heavy metal ions and to improve the application. In this scenario, the Langmuir and Freundlich models are typically used. Table 1 shows the adsorption of metals through a geopolymer and Table 2 represents adsorption of dyes in different types of geopolymers. Ge et al. [164] also showed that when MK-based porous geopolymer spheres were employed to remove Cu^2+^ the Langmuir model fit better than the Freundlich model. These data imply that Cu adsorption by FA-based standard geopolymers is monolayer. In the instance of Pb^2+^ removal, FA-based traditional geopolymers and found that the Langmuir model is more suited to represent the adsorption process than the Freundlich model [26]. As a result of the superior match of the Langmuir model seen in most studies, it is possible to conclude that heavy metal ion adsorption onto the geopolymer adsorbent is classified as monolayer. The interaction between Cu^2+^ and FA-based conventional geopolymer within a temperature range of 25–45℃ using both the Langmuir and Freundlich models and found that both models had a good correlation coefficient, however the Langmuir model fit better [164]. The removal effectiveness of Ni^2+^ using LD-slag based traditional geopolymer and discovered that the Langmuir model suited better than the Freundlich model, implying that the adsorption is monolayer adsorption [152]. On the other hand, classified the adsorption process based on the kind of bond produced, which are physisorption and chemisorption [215]. The former is preferable because the weak Van der Waal force allows for easy renewal of geopolymer as an adsorbent by simple or steam washing, chemical or thermal treatment. It has been observed that after 6–10 consecutive times of use, the adsorption capacity of geopolymer was lowered by roughly 1–10%. The latter, on the other hand, was observed to occur in the majority of the investigations, which is related to the fitting of the Langmuir isotherm. Fourier-transform infrared spectroscopy (FTIR) was used to understand the adsorption mechanism at the microscopic level, despite the fact that very little literature was available. Typically, wavenumbers ranging from 400 or 450 cm^−1^ to 4000 cm^−1^ were used. Furthermore, no significant change in the absorption band of Al–O–Si bending vibrations and Si–O–Si bending vibrations was seen following heavy metal adsorption [169]. However, following Mn^2+^ and Cu^2+^ adsorption, the adsorption band of H–O–H bending vibration at 1647 cm^−1^ was relocated to 1637 cm^−1^, and the peak at 1450 cm^−1^ was definitely shifted to 1431 cm^−1^ after Cu^2+^ adsorption. These spectral changes suggested that when Mn^2+^ and Cu^2+^ were attached to MK-based conventional geopolymer, a complexion with –OH groups occurred. When Ni^2+^ was adherent to LD-slag based conventional geopolymer, the peak of the spectra at 963 cm^−1^ got broader, and postulated that this was due to the formation of a metal ion layer surrounding the LD-slag based conventional geopolymer matrix. Another tiny signal was found at 1447 cm^−1^, showing that heavy metal ions may be chemically bound to the LD-slag-based conventional geopolymer matrix [152].

## 7. Pros and Cons of the Main Conventional Methods Used for the Treatment of Polluted Industrial Wastewater

### 7.1. Electrochemical Coagulation

Pros

Electrocoagulation is a simple procedure. Because it has few moving components, it may be remotely monitored while requiring less monitoring and maintenance. If necessary, the method may usually be altered to accept varying numbers of particles with little effort. In addition, the EC procedure may target several pollutants with a single system and, in certain situations, a single treatment pass. Because it does not include usual chemical additives, it creates lower amounts of sludge that are normally non-hazardous, quickly dewatered, and less expensive to process and dispose of.

Cons

An EC system may require the addition of acids or bases to change the pH, hence it is not totally additive-free. Furthermore, because of the nature of the process, the electrodes are sacrificial and will corrode with time, necessitating their replacement. It can clean plates using a clean-in-place (CIP) procedure that includes acid in the cleaning cycle. The nature of the procedure necessitates the use of electricity as well. While it may not need much at once, power may be more expensive in various parts of the world, which might boost running costs.

### 7.2. Chemical Coagulation

Pros

The fundamental reason for using chemical coagulation is that it shortens the time it would take for the solids to settle on their own. As a result, the overall detention time of the wastewater treatment process is reduced. Chemical coagulation can also help finer colloidal particles and mineral impurities settle. These particles may not settle during the sedimentation process and instead travel through a filtering system.

Cons

Chemical coagulation is fundamentally an additive process. It can lower the number of solids in a solution, but it still requires the addition of chemicals to do so. Adding these ingredients can be difficult and time-consuming, necessitating considerable jar testing. The doses must be quite precise in order to process the influent optimally. Dosage may need to be adjusted on a constant basis because to the fluctuating composition of the wastewater source. The addition of chemicals also leads in the development of a considerable amount of sludge, which must be treated and disposed of after treatment. Because of the nature of the elements included, this sludge is also dangerous. Because sludge cannot be easily dewatered, its bulk and toxicity can push up disposal prices.

### 7.3. UV Disinfection

Pros

Because UV disinfection is a completely physical process, there are no toxic chemicals to deal with. There are no potentially dangerous leftover byproducts in the treated water. It is extremely efficient against the majority of viruses, bacteria, spores, and cysts and requires less contact time than other tertiary wastewater treatment procedures. Furthermore, it has a small footprint for its disinfecting capabilities.

Cons

High quantities of total suspended solids (TSS) can render light inefficient for decontaminating a solution. If the preceding treatment technique is effective in removing TSS, this is a non-issue. Low UV light dosages may be ineffective against certain viruses, spores, and cysts, necessitating longer contact durations or higher-intensity exposure. There is also the possibility of photoreactivation in microorganisms, in which the organisms repair themselves after treatment if the UV dosage is insufficient.

### 7.4. Chlorine Disinfection

Pros

Chlorine is quite cheap and widely available. Furthermore, because it is such a strong oxidizing agent, it may be highly successful at turning huge numbers of hazardous germs inert with a sufficient reaction time.

Cons

Chlorine is very flammable and can produce disinfection byproducts (DBPs) that are hazardous to humans, animals, and aquatic life. It must be handled with care in order to be delivered, stored, and utilized securely. Chlorine disinfection has no effect on viruses, Giardia lamblia, or Cryptosporidium.

## 8. Challenges and Future Perspectives

Despite the fact that several studies have shown that geopolymer can be a possible alternative adsorbent to the existing commercial absorbent, there are certain limitations that need to be addressed further. The first source of concern is a scarcity of raw resources [264,265,266,267]. A number of aluminosilicate materials and industrial wastes can be utilized to make geopolymers [268]. In this regard, the physiochemistry features of those materials often vary, which makes ensuring the quality of geopolymer generated problematic [269,270,271,272,273,274]. Aside from that, the strength of the geopolymer is a source of worry. It is critical for geopolymer adsorbents, particularly those in spherical and monolith forms, to be strong enough to keep their shape when used. The compressive strength of geopolymer changes with the addition of heavy metals, and these variations are difficult to anticipate. Use of chromium oxide or an element of chromium as a filler resulted in a more compact matrix, which has a significant impact on the compressive strength of geopolymer. Furthermore, adding PbO boosted compressive strength whereas adding PbSO_4_ and PbS compounds lowered compressive strength. The following are some drawbacks of the geopolymer production method—Alkali solution readily rusted vessels; there is no single standard for the beginning material for geopolymer production, limiting the larger scale application of geopolymer in industry; and; the complexity of alkaline activator preparation in real conditions. Furthermore, the majority of the current research was conducted on manufactured wastewater that only included a single type of heavy metal ion, which could not accurately represent actual wastewater containing many types of contaminants. Utilizing actual wastewater and discovered a decrease in removal efficiency when compared to the experiment using generated wastewater. This might be attributable to competition between heavy metal ions and other cationic species present in the solution. As a result, more research on real-world wastewater should be conducted in the future to gain a better understanding of the competition between different types of contaminants on the removal effectiveness of geopolymer. As a result, more research into the ability of geopolymers to remove additional types of heavy metal ions and pollutants should be conducted. It is also advised that significant research be conducted on the interaction mechanism of geopolymer with heavy metal ions, particularly at the molecular level, to offer a better understanding and help in the construction of a higher performance geopolymer adsorbent. These are some of the major problems that are limiting larger-scale study and implementation of geopolymer adsorbents. As a result, these characteristics should be thoroughly investigated in order to expand the body of knowledge of geopolymer adsorbents for more stable and promising applications.

Geopolymers have a high potential utility in environmental remediation technology. Its high flexural strength, low carbon footprint, quick curing time and solidification, and strong fire and toxin resistance open up new avenues for researchers. When developed properly, it has the potential to outperform activated carbon and zeolites in the adsorption of hazardous chemicals in both air and water contaminated environments. This is owing to the high porosity of the material. The adsorption effectiveness of geopolymer is determined by the raw ingredients employed in its manufacture. Waste water may be readily transformed to clean water by using geopolymer as a purifier with the right precursors and synthetic techniques. Furthermore, geopolymers can serve as effective photocatalyst supports owing to their unique composition, which yields a variety of metal oxides. However, further research is needed to develop scalable systems that use geopolymers as adsorbents and photocatalyst supports. Surface nano structuring, coating, and composites synthesis are projected to be important undertakings in the near future toward this objective. In all of these scenarios, synthesis and process controls will be important to optimize. Geopolymers are typically found to be highly successful in the removal of cationic chemicals, however it is critical to assess the removal efficiency towards anionic contaminants prevalent in waste water systems. Different raw materials-based geopolymers with different activators should also be tested on nuclear wastes with multicomponent systems in continuous operation to assess the sequestration mechanism of low and intermediate level toxic nuclear materials, as well as their selectivity based on electrostatic/ionic bonding with geopolymer charged sites. Geopolymeric materials can be recommended to develop a socio-economic assessment framework including socio-environment, climate smart, and resilience aspects of global sustainability and innovative engineering for the progressive development of civil infrastructure and society based on the concept of sustainable geotechnical engineering. Previous authors substantiated the sustainable concept of geopolymers by calculating the global carbon budget, oligomer synthesis life cycle assessment, and total energy need for the synthesis cycle. Several research have stated that the geopolymer’s low thermal conductivity and greater durability may be adjusted based on the low operational cost/total energy budget and environmental impact evaluation. The chemical composition of the starting materials and other synthesis factors/designs such as temperature and/or humidity determine the reaction pathways and formation of cross-linked microstructure monomers for the rapid and sustainable polymerization of geopolymers, which are commonly used in civil and geotechnical engineering. This will assist various industries including the environment and ecology in operating under economically feasible and viable operational circumstances in order to maintain with little economic pressure in the new era of sustainability.

## 9. Discussion

In this day and age, on the one hand, water pollution has emerged as the most critical titanic global challenge, and on the other hand, the global paradigm is moving from a linear to circular economy is proving to be quite significant. The valorization of diverse and profound wastes of dissimilar origin is one of the pillars of this novel concept. The innovative geopolymer technology facilitates the application of numerous waste streams as either a precursor or as add-on materials, such as fly ash (FA) and glass waste [272,273,274], which reduces the consumption of natural mineral resources, such as metakaolin (MK), besides evident eco-pros. These assortments of wastes have raised great concerns vis-à-vis their management strategies. Most commonly, these solid wastes are dumped in open spaces, which may otherwise be valuable for other industrial or agricultural activities, converting them into landfills that are accountable for pollution of the environment, surface and subsurface waters, soils and more dangerously for health hazards. This is highly unsustainable in following the concept of the circular economy. That is why the incorporation of the geopolymeric-adsorbent production for water and wastewater treatment will surely reduce not only landfilling practices but also extend systematic waste management, helping to shift from a linear to a circular world economy. Table 3 display the geopolymers application in water and wastewater treatment.

As a fact, water is crucial for life, yet humans trash it anyway, which should be avoided and should purify it to make it reclaimed for reuse. Internationally, around 80% of wastewater is dumped back into water bodies without treatment. The burgeoning universal population urbanization, industrialization and laxity in the correct use of water resources have led to the challenging pollution of freshwater resources. In accordance with the WHO and the United Nations Children Fund, roughly 2.5 billion citizens do not have access to safe potable water to drink, which is a basic necessity to keep one’s self alive next to oxygen to breathe. More or less, 245,000 km^2^ of marine eco-systems are destroyed on account of the discharge of untreated wastewater into seas and oceans. A variety of organic and inorganic contaminants in nature are found mixed with water, and some of them are deeply persistent, noxious and causing loads of deadly diseases, viz., cancer, due to the presence of heavy metal ions. Therefore, getting access to clean water from the purification of wastewater is one of the best strategies to deal with the water crisis. 

Until now, quite a lot of scientific investigations and approaches, such as conventional coagulation, ion exchange, adsorption, chemical precipitation, electrolysis, reverse osmosis, electro-dialysis, are being brought to play for their eliminations from wastewater in order to conserve it as a freshwater resource. However, it seems that the conventional methods are in short supply to cope with the existing gigantic dilemma of wastewater treatment. The novel geopolymer composites are found interesting, which are emerging as the adsorbent material of choice, not only because of their surface area criterion but also for their functioning, as well as chemical accessibility. Furthermore, geopolymer composites exhibit versatile and enhanced properties, such as corrosion resistance, high specific stiffness, thermal insulation, low density, specific strength and great absorbance quality for heavy metal ions. Furthermore, adding-on various waste materials for obtaining brand-new GP materials is possible.

## 10. Conclusions

The present review drives to conclude that “Clean water is a human right” and there must be alternative water resources for a step forward towards “Zero water crisis”. With a view to having pure potable drinking water for all the human beings and lives on the planet, the approach of water and wastewater treatment is highly essential to hit the goal of the reuse of reclaimed water. The diverse methods to clean water by removing impurities of extremely perilous and noxious heavy metal ions are widespread for wastewater treatment; however, geopolymer technology seems to be highly appreciated and promising through its adsorbent composites. For that reason, significant enhancement and interest have been taken during the last decade in this regard. Revolutionarily, the geopolymer composites came forward and became known, gaining tremendous popularity as heavy-ion adsorbents on account of copious valuable attributes, especially of mechanical strength, porosity, durability, ion-exchange competence, etc., proving them superior to other competing materials. 

Geopolymerization, the process of production of geopolymer composite adsorbents, is quite simple with low-energy and low-temperature requirements and significantly mitigated carbon footprints. Commercially, the shift of a linear economy into a circular one is possible with geopolymer composites due to their readiness to incorporate diverse wastes of dissimilar origin with them as precursors or industrial wastes bringing not only their systematic waste management into play but also heading lucratively towards cost-effectiveness. This cutting-edge technology has evidently revealed a promising potential for heavy-metal removal from water and wastewater with simultaneous recovery of different metals, rare earth elements, ammonium, sulfate, organic dyes, etc., boosting its profitability. 

The research studies throwing light on their successful application as catalysts or catalyst supports related to the use of geopolymer composites point towards their great potential for photo-catalytic degradation of recalcitrant organic compounds as micro-pollutants. From the standpoint of techno-applications; the key magnetism of geopolymers arrives from the actuality that their production is readily scalable, and the process of production is rendered as “Green” which brings about enormous mitigation of wastes enabling cleaner production systems in the manufacturing industries. Nevertheless, it is noteworthy that the surface redox reactions, in general, and origins of photo-activity mostly stay open issues with respect to geopolymers. While geopolymer composites have already made it to numerous commercial applications, their fundamental surface chemistry and typical chemical structure will persistently offer opportunities to explore them further. Geopolymeric membranes exploit the built-in mesoporosity of the materials, making their application fit for micro or ultra-filtration. Their extensive use for water and wastewater treatment include lower pressure filtration media, water treatment residues, such as various sludge and spent adsorbents, pH buffering materials, anti-microbial materials, carrier media for biological treatment processes. Although a lot of successful work is found on the adsorption properties and performances of geopolymers in the reviewed utilizations that exhibits absolute potential, still more, advanced studies are essential on a large scale and for a long duration to establish the techno-scientific readiness of them fully in order to prove them totally promising materials to extend confidence to industries, engineers and field workers. Financially, the advantages need to recover valuable metals, nutrients, higher energy products from side streams, incorporation of wastes, etc., and will surely bring about a circular economy towards the next echelon. Interestingly, it is also possible to cover the costs of the wastewater treatment course of action from the revenue income of eliminated materials, instituting a prominent position for them.

The cheapest and newest method of geopolymer technology will promote the reuse of the treated effluent for a variety of purposes, such as agriculture. Geopolymers are found far above the ground affinity for NH_4_^+^ ions as the charge-balancing Na^+^ cations in their alumina-silicate network. Feasibly, the porous geopolymer components produced by means of direct ink writing technique of 3D printing for the exclusion of NH_4_^+^ ions from wastewater are found to be very significant. The printed geopolymer adsorbents demonstrate excellent cation exchange capacity with NH_4_^+^ subtraction efficiency. The admirable capacity of 3D-printed geopolymers for brilliant ion exchange is assigned to their higher mechanical properties and elevated permeability. That means printed geopolymer composites are more efficient than synthetic zeolites and conventional ceramics because of their lesser energy-intensive production at a low temperature using locally profound wastes. The synergy between material and geopolymerization contributes to the effectual customization and tailoring of adsorbents with optimal porosity, mostly favoring permeability and optimum mechanical properties.

## Figures and Tables

**Table 1:** Adsorption of metals through a geopolymer.

Source Material for Geopolymer	Adsorbate	Alkaline Activator	Adsorption Capacity (mg/g)	References
Metakaolin, Rice Husk Ash	Crystal Violet	KOH	276.9	[163]
Fly Ash	Cd^2+^	NaOH, Na_2_SiO_3_	9.02	[246]
Pyrophyllite	Cd^2+^	NaOH	7.82	[247]
Pyrophyllite	Co^2+^	NaOH	7.1	[247]
Metakaolin	Co^2+^	NaOH, Na_2_SiO_3_	69.23	[169]
Metakaolin	Mn^2+^	NaOH, Na_2_SiO_3_	72.34	[169]
Pyrophyllite	Ni^2+^	NaOH	7.28	[247]
Pyrophyllite	Pb^2+^	NaOH	7.54	[247]
Ld Slag	Zn^2+^	NaOH, Na_2_SiO_3_	86	[248]
Fly Ash, Blast Furnace Slag	Cs^+^	NaOH,	15.24	[156]
Metakaolin	Cu^2+^	KOH, silica fume	40	[249]
Metakaolin	Cu^2+^	NaOH, Na_2_SiO_3_	62.5	[250]
Metakaolin	Ni^2+^	NaOH, Na_2_SiO_3_	42.61	[169]
Blast Furnace Slag	SO_4_^2−^	NaOH, Na_2_SiO_3_	119	[113]
Metakaolin	Cd^2+^	NaOH, Na_2_SiO_3_	98.10	[174]
Metakaolin, Clinoptilolite	Cr^3+^	NaOH, Na_2_SiO_3_	21.84	[174]
Fly Ash, Iron Ore Tailings	Cu^2+^	NaOH, Na_2_SiO_3_	113.41	[103]
Metakaolin	Cu^2+^	NaOH, Na_2_SiO_3_	44.73	[174]
Metakaolin, Clinoptilolite	Pb^2+^	NaOH, Na_2_SiO_3_	261.22	[174]
Volcanic Tuff	Zn^2+^	NaOH	14.83	[251]
Metakaolin, Clinoptilolite	Zn^2+^	NaOH, Na_2_SiO_3_	35.88	[174]
Metakaolin, Al_2_O_3_	Methylene Blue	H_3_PO_4_	4.26	[110]
Metakaolin	Ca^2+^	NaOH	24	[173]
Fly Ash	Cu^2+^	NaOH	152.3	[27]
Metakaolin	Cu^2+^	NaOH	34.5	[173]
Metakaolin	Pb^2+^	NaOH	45.1	[173]
Metakaolin	Cu^2+^	NaOH, Na_2_SiO_3_	52.63	[164]
Fly Ash	Methylene Blue	NaOH, Na_2_SiO_3_	50.7	[188]
Metakaolin	NH_4_^+^	NaOH, Na_2_SiO_3_	21.07	[165]
Blast Furnace Slag	As(III)	NaOH, Na_2_SiO_3_	0.52	[107]
Fly Ash	Co^2+^	NaOH, Na_2_SiO_3_	66	[105]
Fly Ash	Co^2+^	NaOH, Na_2_SiO_3_	59	[252]
Fly Ash	Co^2+^	NaOH, Na_2_SiO_3_	52	[252]
Fly Ash	Cu^2+^	NaOH, Na_2_SiO_3_	77	[105]
Blast Furnace Slag	Ni^2+^	NaOH, Na_2_SiO_3_	4.42	[107]
Fly Ash	Pb^2+^	NaOH, Na_2_SiO_3_	118.6	[102]
Fly Ash	Pb^2+^	NaOH, Na_2_SiO_3_	6.34	[1]
Blast Furnace Slag	Sb(III)	NaOH, Na_2_SiO_3_	0.34	[165]
Metakaolin	NH_4_^+^	NaOH, Na_2_SiO_3_	32	[192]

**Table 2:** Adsorption of dyes in different types of geopolymers.

Geopolymer as a Adsorbent	Dye	Adsorption Capacity (mg/g)	Efficiency Degradation (%)	References
TiO_2_ geopolymer composite	MB	20.11	97	[253]
Phosphoric acid-based geopolymer	MB	3.01		[110]
Fly ash geopolymer	MB	37.04	–	[254]
Metakaolin-based geopolymer	MB	43.48	-	[255]
Magnetic geopolymer	AR97	1814.27		[256]
Geopolymer	CR	–	100	[257]
Fly ash-based geopolymer	BY	36.364		[258]
Magnetic geopolymer	AG	183.17		[259]
Metakaolin-based geopolymer	MV10B	276.9		[163]
Metakaolin geopolymer	MO	0.333	–	[260]
Magnetic geopolymer	PR	39.21		[259]
Alkali-activated phosphorous slag	BV	46.58		[261]
Fly ash geopolymer	MB	–	92.79	[8]
Alkali-activated phosphorous slag	MGO	46.36		[261]
Magnetic geopolymer	AG16	400		[262]
Geopolymer	MV	–	91.16	[263]

Methylene blue—MB, Basic yellow 2—BY, Acid green—AG, Procion red—PR, Basic violet—BV, Malachite green oxalate—MGO, Acid green 16—AG16, Methyl violet 10B—MV10B, Acid red 97—AR97, Methyl orange—MO, Crystal violet—CV, Congo red—CR, Methyl violet—MV.

**Table 3:** Geopolymers application in water and wastewater treatment.

Geopolymers Application in Water and Wastewater Treatment	Geopolymer Source Materials	References
Removal of Adsorption and organic pollutants	FA	[275]
Removal of Air particulate matter	MK	[276]
Antimicrobial and membrane filtration	MK	[277]
Desalinization by pervaporation	MK + HZ	[236]
	AS	[278]
Pervaporation	MK	[279]
	GGBFS	[135]
	AS	[276]
	L	[280]
Removal of Heavy metals	MK	[85]
	FA	[281]
	MK + F	[282]
Household wastewater treatment	FA	[283]
Method of Ion exchange	MK + F	[237]
	MK	[284]
Method of Oil separation	FA	[285]
	FA	[286]
	FA + B	[287]
	FA + Q + C	[288]
	GGBFS	[233]
Removal of Organic pollutants	MK + FS	[289]
Textile wastewater treatment	FA	[290]
Method of Green liquor treatment	MK	[291]
Method of Water desalination	MK	[232]
	MK + FS	[292]
	FA	[293]
	MK	[294]
Removal of turbidity	MK	[130]
Method of Oil separation from Removal of organic pollutants	MK	[295]

FA—Fly ash; MK- Metakaolin; HZ—Hydroxysodalite zeolite; AS—Aluminium-silica powder; GGBFS—Grand Granulated Blast furnace slag; L—Laterite; F—Fumed silica; B-Bauxite; Q—quartz; C—calcium carbonate.

## Data Availability

Data is contained within the article.
